# IgA-mediated control of host-microbial interaction during weaning reaction influences gut inflammation

**DOI:** 10.1080/19490976.2024.2323220

**Published:** 2024-03-04

**Authors:** Wenjie Tang, Yusen Wei, Zhixiang Ni, Kangwei Hou, Xin M. Luo, Haifeng Wang

**Affiliations:** a College of Animal Science, Zhejiang University, Hangzhou, China; b Department of Biomedical Sciences and Pathobiology, Virginia Tech, Blacksburg, VA, USA

**Keywords:** Weaning reaction, gut inflammation, bacteroides uniformis, CD138^+^ plasmacyte, IgA-coated bacteria

## Abstract

The mechanisms of how host-microbe mutualistic relationships are established at weaning contingently upon B-cell surveillance remain inadequately elucidated. We found that *CD138*^+^ plasmacyte (PC)-mediated promotion of IgA response regulates the symbiosis between *Bacteroides uniformis* (*B. uniformis*) and the host during the weaning period. The IgA-skewed response of *CD138*^+^ PCs is essential for *B. uniformis* to occupy a defined gut luminal niche, thereby fostering stable colonization. Furthermore, *B. uniformis* within the natural gut niche was perturbed in the absence of IgA, resulting in exacerbated gut inflammation in IgA-deficient mice and weaned piglets. Thus, we propose that the priming and maintenance of intestinal IgA response from *CD138*^+^ PCs are required for host-microbial symbiosis, whereas the perturbation of which would enhance inflammation in weaning process.

## Graphical abstract

**Figure:**
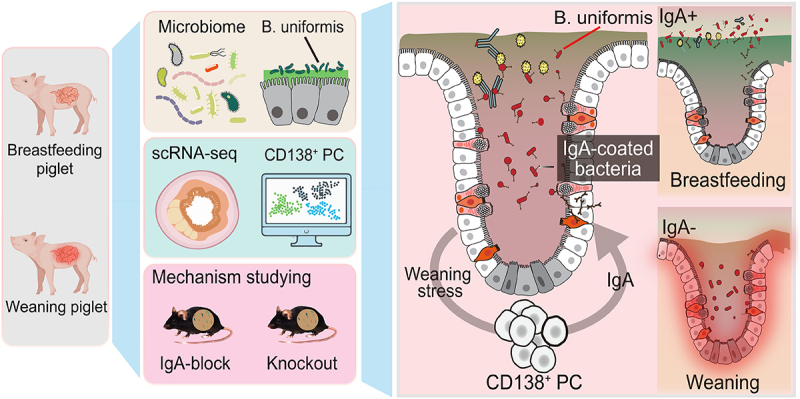

## Introduction

Neonates acquire various protective immunoglobulins through the transplacental transfer and maternal milk uptake during lactation. This so-called passive immunity provides protection from pathogenic infections and tolerance to colonizing commensal microbes in the gastrointestinal mucosa.^1,2^ At weaning, a window of opportunity arises for commensal bacteria to colonize, which participate in the maturation of the immune system.^3^ Disruption of the microbiota during weaning may perturb the intestinal immune homeostasis, leading to defects in protective immunoglobulin responses later in life.^4^ Following weaning, the protective force dissipates, rendering young mammals susceptible to gastrointestinal infections, particularly when the host-microbe mutualism fails to establish.^5,6^ At this phase, antibody production from B cells in response to the microbiota is a critical event in early-life immune response, with antibodies mediating agglutination toward colonizing microbes and potentially conferring defense against invading microbes.^7^ IgA is the predominant antibody isotype present at intestinal mucosal surfaces and plays a critical role in mediating the coordination between immune and microbial interactions.^8,9^ During bacterial infections, IgA promotes intestinal pathogen clearance by crosslinking the bacteria to prevent encroachment on the lumen surface neutralizing bacterial toxins, and/or modulating bacterial metabolic activity.^10,11^ Additionally, recent studies have demonstrated that most indigenous members of bacterial taxa in the gut can stimulate IgA production to varying levels and then be bound by IgA.^12,13^ Thus, IgA is crucial to protecting gastrointestinal mucosa from pathogenic attack, while at the same time benefits commensal species by establishing strong host-microbial symbiosis. If IgA-mediated regulation of mucosal colonization of commensal bacteria is disrupted during early life, it is likely that these bacteria that would otherwise colonize harmoniously would induce immune abnormalities.^12,14,15^

The cessation of breast-feeding and dietary shifts can alter gut microbial ecology of infants, resulting in a feed-oriented composition enriched in *Bacteroides*.^16^ A recent study demonstrated that Bacteroides were the primary drivers of phenotype variation in the gut-associated B cell repertoire, and their presence and subsequent IgA response would shape host-commensal interaction in mice.^17^ However, few studies have investigated Bacteroides-immune mutualism during the weaning period, which, if perturbed, might lead to an increase of intestinal disease susceptibility. It also remains unclear whether Bacteroides possess symbiotic or disease-triggering properties while specific B cell subsets in the gut tissue undergo class switching to IgA-secreting cells. Due to ethical constraints and methodological limitations that preclude direct clinical investigation of weaning stress in infants, neonatal animal models, such as piglets, represent a crucial tool for exploring the intestinal immune response provoked by the intestinal microbiota during weaning.^18^ Indeed, Piglets as a weaning model thus holds promise for facilitating the translation to human infants in terms of gut development and clinical management of enteric disorders.^19,20^ We used the weaning piglet model combined with knockout mice to investigate the responsiveness of IgA-producing B cells to bacterial exposure in the intestinal mucosa during the weaning period.

## Results

### Weaning induces decreased gut immune barrier function and an increase of IgA-coated bacteria

**Figure 1.**
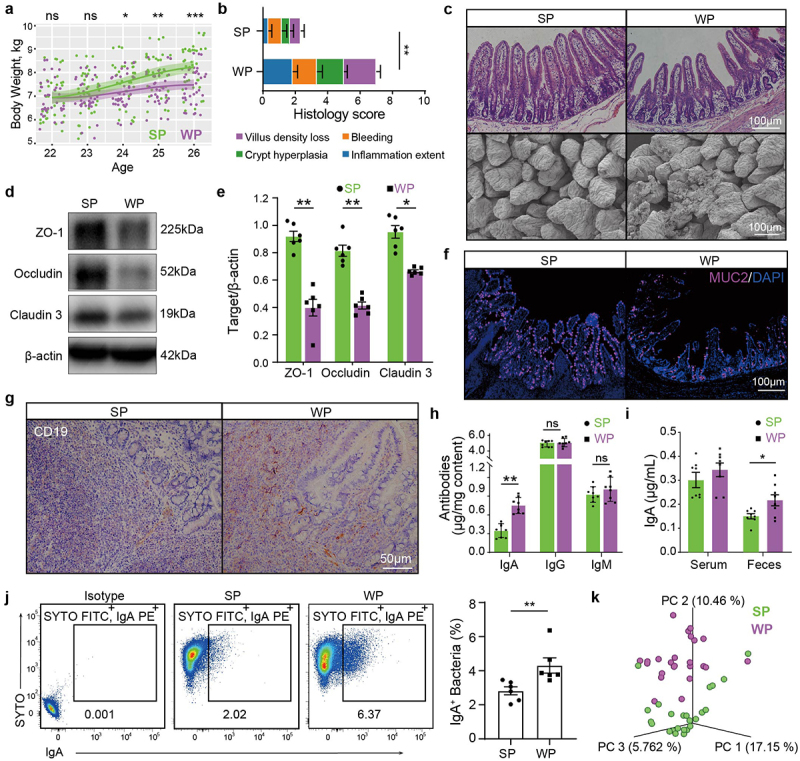
Weaning induces decreased gut barrier function and an increase of IgA-coated bacteria.

Weaning imposes sudden and simultaneous stressors on piglets, ultimately resulting in suboptimal weight gain (Figure 1a). Inflammation exists in the ileum and colon, with a remarkably higher histological score in the weaned piglets (WP) than in the suckling piglets (SP) (Figure 1b, c and Supplementary Fig. S1a). We examined several major indicators of intestinal physical and immune barrier functions, and all were significantly lower in the WP group (Figure 1d–f and Supplementary Fig. S1b, c). B cells are present in the lamina propria of the intestinal mucosa in the SP group, but breached the epithelium in the WP group (Figure 1g and Supplementary Fig. S1d). The levels of IgA, but not IgG or IgM, in intestinal tissues were significantly higher in piglets subjected to weaning treatment compared to those in breast-feeding (Figure 1h), suggesting IgA as the primary immunoglobulin participating in B cell immune response during the weaning period. In addition, IgA levels were found to be elevated in the fecal samples of the WP group but not in their sera (Figure 1i). Notably, IgA secreted across the mucosal surface is both induced by and reactive to the gut microbiota.^21,22^ By subjecting fecal samples to IgA staining and analyzing them via flow cytometry,^9,23^ we confirmed the presence of IgA-coated bacteria in WPs (Supplementary Fig. S1e). A significant increase was found in the proportion of IgA-positive bacteria in WPs compared with those in SPs (Figure 1j). Furthermore, we observed discernible dissimilarities in microbial communities between the two groups by utilizing 16S rRNA sequencing profiles (Figure 1k). These results indicate that the gut barrier function is impaired upon weaning, allowing B cells to penetrate the gut epithelium where they produce more IgA to coat gut bacteria.

a, Body weight of piglets in SP and WP groups over 7 days (*n* = 32). b, Comparison of the total histology scores and individual histological criterion scores between the two groups (*n* = 6). c, Ileum morphology visualization via H&E-stained sections and scanning electron microscopy in SP and WP groups (scale bars, 100 µm). d and e, Assessment of intestinal tight junction protein expression by western blotting (*n* = 6). f, IF staining for goblet cells (MUC2, pink; scale bars, 100 µm). g, Visualization of *CD19*^+^ B cells in intestinal tissue via representative IHC images. h, Intestinal tissue IgA, IgG, and IgM levels analysis via ELISA (*n* = 6). i, Serum and stool IgA analysis via ELISA. j, Flow cytometry analysis of fecal bacterial IgA binding in SP and WP groups, with quantification of IgA-bound fraction (*n* = 6). k, PCoA emperor plot reveals separation between SP and WP microbiomes (*n* = 24). Statistical analysis: unpaired *t*-test. *p < 0.05, **p < 0.01, and ***p < 0.001.

### The *Bacteroides* taxon is enriched upon weaning and associated with diarrhea when not highly coated with IgA

**Figure 2.**
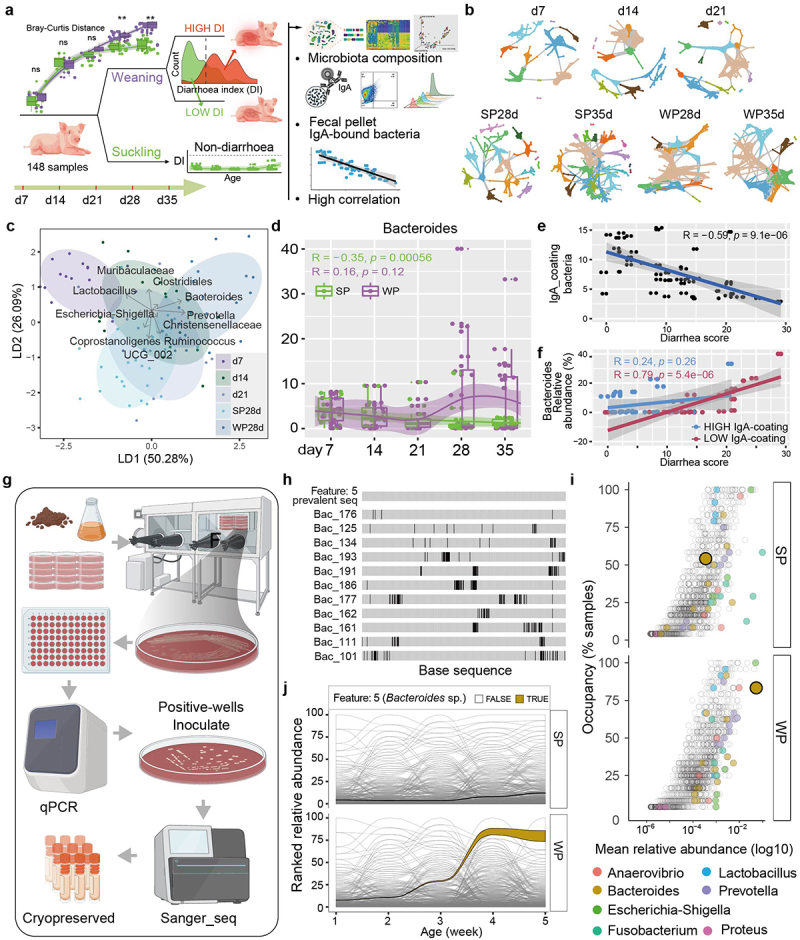
The bacteroides taxon is enriched upon weaning and associated with diarrhea when not highly coated with IgA.

Longitudinal analysis of gut microbiota profiles was performed for 144 stool samples from birth to 2 weeks post-weaning using 16S rRNA sequencing (Figure 2a). An average of 52,337 ± 7286 features was generated per sample, from which a total of 956 features were identified with a relative abundance of more than 0.01%. A progressive change was observed in alpha diversity with the development of piglets, which persisted to remain similar to the sow’s microbiota (Supplementary Fig. S2a). However, weaning led to an obvious reduction in alpha-diversity, which not only fell below their suckling counterparts but also remained lower than the sow. The Bray-Curtis dissimilarity analysis unveiled a marked alteration in the composition of the gut microbiota throughout the diverse developmental stages and a strikingly discrepancy between the SP and WP groups (Supplementary Fig. S2b).

a, Illustration depicting the longitudinal study design encompassing collection of fecal samples, gut microbiota analysis, subject grouping, and correlation analysis. b, Visualization of molecular ecological networks spanning from birth to weaning. Diverse color-coded large modules, consisting of ≥3 nodes, with smaller modules displayed in gray. c, Linear discriminant analysis (LDA) was employed to discern the variation in piglet microbiota composition over time. The length and direction of the arrows depict the normalized scaling for each genus. d, Relative abundance of the Bacteroides in SP (green) and WP (purple) groups. Adjusted R and *p* values were derived from linear regression analyses. e, The percentage of IgA-coated bacteria was correlated by linear regression with diarrhea score in weaned piglets (*n* = 48). Linear regression and Pearson’s correlation coefficient were shown. f, The relative abundance of the Bacteroides was positively correlated by linear regression with diarrhea score in group with low levels of IgA-coated bacteria, whereas no correlation was observed in group with high levels of IgA-coated bacteria (*n* = 24). Linear regression and Pearson’s correlation coefficient were shown. g, WP were subjected to the isolation and characterization of gut bacterial species, employing a combination of qPCR screening, custom anaerobic medium cultivation, and Sanger sequencing techniques. h, Alignment of 16S rRNA sequences of each isolate to the Bacteroides sp. (Feature ID: 5) identified from *Silva* database. Black lines indicate mismatch. i, Occupancy–abundance curves for the SP and WP communities. Bacteroides sp. features detected as enriched in WPs are highlighted. j, The abundance levels of individual members within the community were ranked over time. The high abundance member of the WP group, Bacteroides sp. (Feature ID: 5), is highlighted.

Time-course analysis revealed distinct developmental trajectories of the four identified phyla Firmicutes, Bacteroidetes, Proteobacteria and Actinobacteria within the gut microbiota during early life and the weaning period (Supplementary Fig. S2c, d). Notably, Bacteroidetes was significantly more prevalent in the WP compared to the SP in 2 weeks following weaning (Supplementary Fig. S2d). Seven time-series molecular ecological networks (Figure 2b) were derived by applying a previously described method.^24^ The co-occurrence patterns under weaning and suckling followed distinct successional trajectories over time, as evidenced by regression analysis of six network topological parameters over time (Supplementary Fig. S2e), which suggests that the gut microbiota established a more complex and stable network in the SP group compared to the WP group. Specifically, Bacteroides was found to occupy a central position in the network structure in WP, while displaying a distinct pattern in SP (Supplementary Fig. S3a). Consistently, Bacteroides was identified as a signature genus in the weaning process by a supervised learning analysis (Figure 2c and Supplementary Fig. S3b). The relative abundances of Bacteroides remained consistently expanded in consecutive time points in the WP group, while remaining low in the SP group (Figure 2d).

Next, we sought to examine whether the differences in Bacteroides abundance were associated with diarrhea-like features under weaning stress. Across all samples, a moderate correlation (*R* = 0.48) was found between the abundance of Bacteroides and incidence of diarrhea (Supplementary Fig. S3c). However, within the WP group, there appeared to be individuals that displayed both low diarrhea incidence and high Bacteroides abundance, although it was unclear whether the bacteria were IgA coated. The levels of IgA-coated bacteria were thus taken into consideration. A significant negative correlation (*R* = −0.59) existed between the abundance of IgA-coated bacteria and diarrhea incidence (Figure 2e). Following these observations, we hypothesized that the binding of IgA to bacteria could potentially curtail the diarrheal impacts of Bacteroides. To verify this, all piglets were re-classified into high or low IgA-coated phenotypes on the basis of their respective levels of IgA-coated bacteria (Figure 2a and Supplementary Fig. S3d, e). In the group with lower levels of IgA-coated bacteria, we found a significant positive correlation (*R* = 0.79) between the abundance of Bacteroides and diarrhea index, whereas no correlation was observed in the group with higher levels of IgA-coated bacteria (Figure 2f). This suggests that without sufficient IgA coating, Bacteroides enriched upon weaning may be pathobionts that are associated with diarrhea.

To enable functional analysis of specific bacterial strains, we devised qPCR-based screening and anaerobic isolation strategies to recover several bacteria with variable abundance in weaned piglets (Figure 2g). Sixty-nine bacterial strains belonging to seven different genera were isolated, including members of the genus Bacteroides. Bacterial isolates were classified based on their genetic similarity to known feature sequences produced by 16S rRNA sequencing. A feature was considered as the representative sequence of the isolated strain if the variation was below 1%, such as Feature 5 for the *B. uniformis* isolate Bac-176 (Figure 2h). Following this, abundance–occupancy curves were generated for bacterial communities in both SP and WP groups. The *B. uniformis* taxon, classified as Feature 5, had significant predominance: not only was it found in most samples, but also its relative abundance was highest among all features in the WP group (Figure 2i). Furthermore, interrogating its temporal dynamics, we found that Feature 5 became the predominant taxon in intestinal communities in the WP group during the weaning period (Figure 2j).

### *Weaning stress remodels epithelial and immune cells to emphasize IgA-producing* CD138*^+^ PCs*

**Figure 3.**
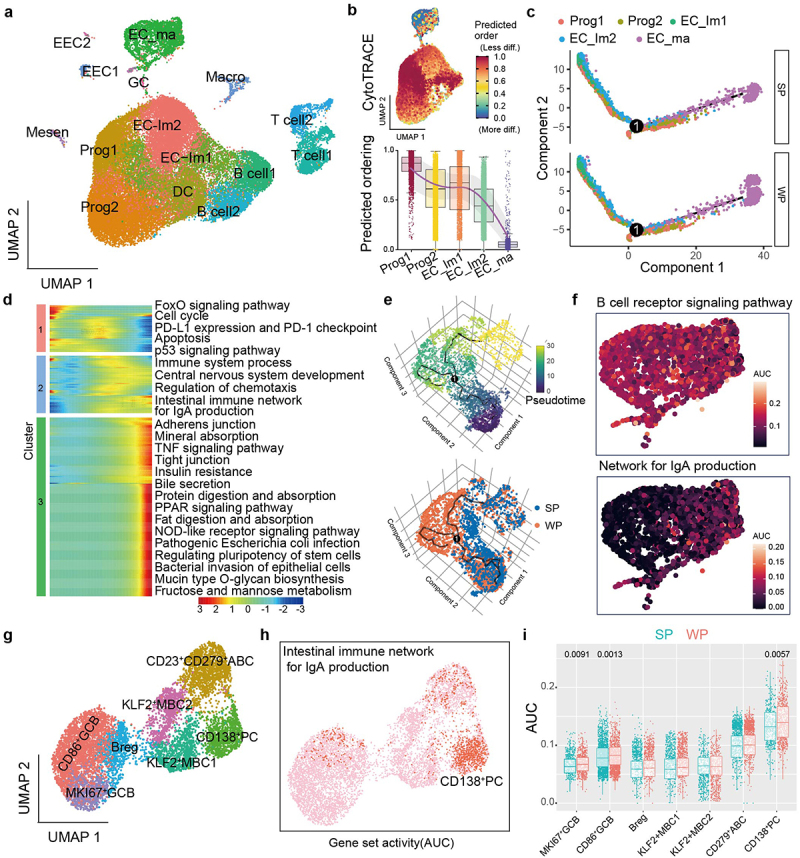
Weaning stress remodels epithelial and immune cells to emphasize IgA-producing *CD138*^+^ PCs.

Droplet-based single-cell RNA sequencing was conducted on cells isolated from intestinal tissues. Transcriptional profiles included more than 42,000 individual epithelial and immune cells, which were subsequently assigned into 15 distinct clusters (Figure 3a). Through clustering analysis according to distinct transcriptional states, eight primary compartments were identified that consist of four epithelial cell lineages: epithelial progenitor cells (*STMN1*, *PCNA*, and *SLBP*), enterocytes (*FABP1*, *FABP6*, and *APOA1*), enteroendocrine cells (*CHGA*, *CHGB*, and *GCG*), and goblet cells (*SPINK4*, *AGR2*, and *FCN2*); and four immune cell lineages: B cells (*CD19A*, *MS4A1*, *CD79A*, and *JCHAIN*), T cells (*CD3E*, *CD3D*, and *GNLY*), dendritic cells (*CCR7*, *KLF2*, and *PLAC8*), and macrophages (*C1QA*, *C1QB*, and *CD68*) (Supplementary Fig. S4a). Based on the cell type annotation, a gene expression matrix spanning progenitor, immature, and mature enterocyte clusters was extracted to predict general differentiation states of enterocytes by running CytoTRACE (Figure 3b). Next, we calculated the sequence of gene expression changes of each cell from all enterocyte clusters within a dynamic differentiae process, and place each cell at its proper position in the transition trajectory. The trajectory of cellular differentiation from progenitor cells to mature enterocytes was found to be globally conserved between the two groups (Figure 3c). During the transition of cells across developmental states, a process of transcriptional reconfiguration occurred whereby certain genes associated with pluripotency and self-renewal properties were downregulated, while others that were crucial for the functional characteristics of enterocytes were activated (Figure 3d). Importantly, we noted that the impact of weaning stress was primarily apparent in the late stages of intestinal epithelial cell maturation (Figure 3c). We thus decided to focus on mature enterocytes. All the enterocytes were ordered and re-clustered in pseudotime using Monocle 3. Interestingly, the reconstructed trajectory of enterocytes exhibited a clear branch point to distinguish the two groups (Figure 3e). A branched expression analysis model was utilized to ascertain genes that exhibited differential expression. Surprisingly, the heatmap showed that gene modules associated with B cell response pathways were significantly upregulated in the WP branch (Supplementary Fig. S4b). AUcell was used to score B cell response-related signaling pathways across all enterocytes. Strikingly, we observed marked upregulation of B cell receptor signaling pathways, as well as an increase in the network for IgA production, in the WP group (Figure 3f and Supplementary Fig. S4c).

a, UMAP visualization of all ileal cells in SP and WP, with each cell colored based on its corresponding cell-type compartment. b, Boxplots showing CytoTRACE values for progenitor cells, immature enterocytes and mature enterocytes c, Differentiation trajectory of progenitor cells, immature enterocytes and mature enterocytes, assigning distinct colors to each cell type for visualization. d, Heatmap presenting the signaling pathways that display significant variations along the pseudotime. e, 3D differentiation trajectories of mature enterocyte, where each cell was assigned a finite pseudotime value. f, UMAP plots of enterocyte cluster overlay for the AUC scores of given genesets. g, UMAP visualization of all B cells in SP and WP, with each cell colored based on its corresponding cell-type compartment. h, UMAP plots of B cell clusters are colored based on whether they pass the AUC score assignment threshold for selected pathway activates. Cells that surpassed the threshold were colored in red. i, Boxplots showing individual cell AUC score for IgA production activity.

To investigate the effects of weaning stress on B cell populations, the original object was subdivided into a B cell-specific subset. Subsequently, unsupervised clustering was performed on the selected populations to unravel their underlying dynamics (Figure 3g). According to the expression levels of canonical marker genes, two clusters of germinal-center B (GCB) cells (*AICDA*, *PAX5*, *BCL6*, and *NUGGC*),^25^ two clusters of memory B cells (*CD11B* and *KLF2*),^26^ a cluster of B regulatory cells (*IL10* and *CD307D*), and a cluster of *CD138*^+^ plasmacytes (PCs) (*CD138*, *CD38*, *BLIMP1*) were identified (Supplementary Fig. S4d).^27^ Based upon our previous identification of enriched pathways associated with B cell responses in enterocyte clusters, we focused on the enrichment analysis of a gene set involved in intestinal immune network for IgA production across all subtypes of B cells. To estimate the proportion of genes in the gene set that were highly expressed in each cell and determine whether the signature of IgA production was active or not, we applied the AUC algorithm and manually selected a threshold (AUC >0.15, Supplementary Fig. S4e). Unexpectedly, the cells with the highest enrichment of the identified pathway were primarily *CD138*^+^ PCs; and significantly higher enrichment scores were found in the WP group than the SP group (Figure 3h, i). These results suggest that the effector functions of *CD138*^+^ PCs during the weaning process may impact the protective responses to enterocytes through the production of IgA.

### B. uniformis *elicits IgA responses in* CD138*^+^ PCs and alleviates DSS-induced colitis*

**Figure 4.**
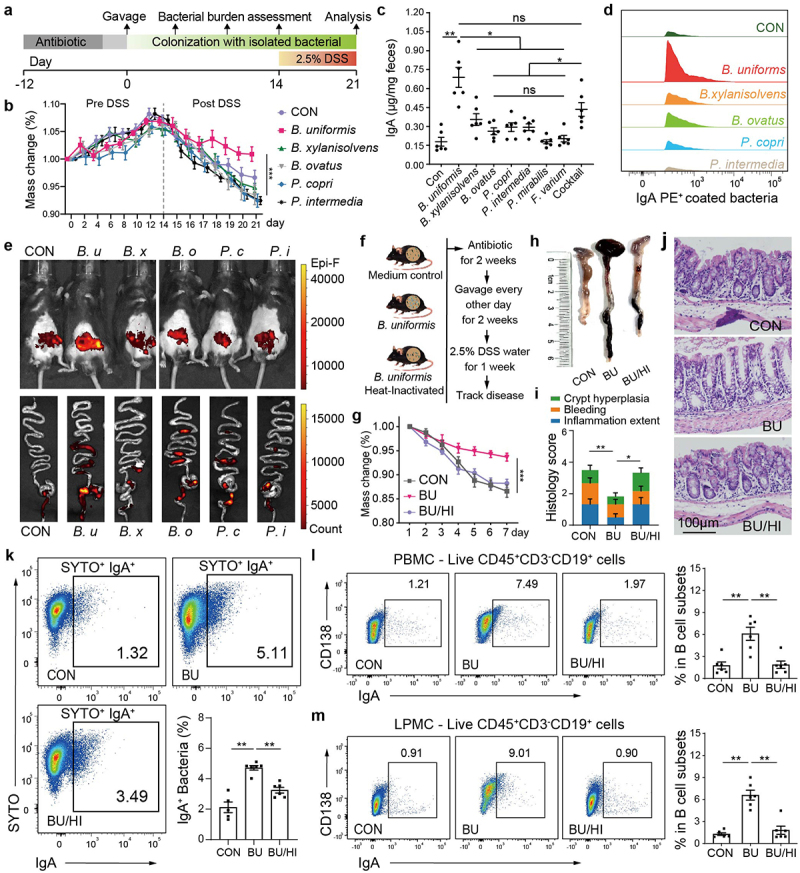
*B. uniformis* elicits IgA responses in *CD138*^+^ PCs and alleviates DSS-induced colitis.

A mouse colitis model was employed to ascertain whether individual gut bacterial isolates elicit a discernible impact on specific B cell responses, skewing them toward IgA production. Prior to performing sequential gavage of individual isolates, the mice underwent a tailored three-course antibiotic conditioning regimen to eradicate endogenous microbiota (Method for details, Figure 4a). The pseudo-germ-free mice were then mono-colonized with different representative bacteria from the prominent genera of the WP group including Bacteroides (*B. uniformis*, *B. xylanisolvens*, and *B. ovatus*), Prevotella (*P. copri* and *P. intermedia*), Fusobacterium (*F. varium*), and Proteus (*P. mirabilis*). The introduction of the bacterial strains to mice prior to DSS intervention did not elicit notable changes in body weight or pathological imprints (Figure 4b), validating the nonpathogenic nature of these strains in wild-type mice under steady-state condition. However, following DSS-induced colitis, gavage of mice with *B. uniformis* resulted in significantly reduced weight loss compared to the control group, whereas other strains did not (Figure 4b). Importantly, *B. uniformis* mono-colonized mice exhibited markedly higher IgA secretion compared to mice colonized with any of the other six bacterial species (Figure 4c). Meanwhile, mice receiving a cocktail of all seven bacterial species produced fecal IgA levels comparable to those mono-colonized with *B. uniformis*. Remarkably, in agreement with the abundance of luminal IgA, mice from *B. uniformis* administrated group exhibited the highest fraction of IgA-coated bacteria than other groups (Figure 4d and Supplementary Fig. S5a). These results suggest that *B. uniformis* is a robust gut IgA inducer and reshapes the ecological niche of intestinal IgA-bound bacteria.

a, Timeline of antibiotic treatment, mono-colonization of individual gut bacteria and DSS-induced colitis in SPF mice. b, Weight change of C57BL/6J mice exposed to piglet isolated bacteria pre- and post-DSS colitis. c, Fecal IgA level in C57BL/6 mice colonized with individual gut-isolated bacteria of piglet (*n* = 6). d, Flow-cytometry analysis of fecal bacterial IgA binding in mice colonized with individual isolates, shown by histogram of IgA-coated bacteria fraction. e, Whole-body imaging and intestines imaging *ex-vivo* assessing the *B. uniformis* burden in intact animals. Mice were administrated with FDAA-labeled *B. uniformis*. f, Mice were gavaged for 2 weeks with medium (control), *B. uniformis*, or heat-killed *B. uniformis* and treated with 2.5% DSS for 1 week. g, Weight change of mice exposed to *B. uniformis* under DSS intervention for 7 days (*n* = 6). h, Macroscopic image of the colon on day 7 and length of colon. i, Comparison of the total histology scores and individual histological criteria scores across groups (*n* = 6). j, Representative micrographs of H&E-stained colonic sections, demonstrating that *B. uniformis* administration results in a reduction in intestinal inflammation compared to both control and heat-killed *B. uniformis*. Scale bar, 100 µm). k, Flow cytometry analysis of fecal bacterial IgA binding in medium (control), *B. uniformis*, or heat-killed *B. uniformis* groups, with quantification of IgA-bound fraction (*n* = 6). l, PBMCs of all mice were collected for flow cytometry analysis of frequency of *CD138*^+^ IgA^+^ among live *CD45*^+^ *CD3*^−^ *CD19*^+^ cells, with quantification of *CD138*^+^ IgA^+^ precent (*n* = 6). m, LPMCs of all mice were collected for flow cytometry analysis of frequency of *CD138*^+^ IgA^+^ among live *CD45*^+^ *CD3*^−^ *CD19*^+^ cells, with quantification of *CD138*^+^ IgA^+^ precents (*n* = 6). Statistical analysis: (i, k, l, and m) One-way ANOVA, (c) Mann–Whitney *U* test or (b, g) Two-way ANOVA. *p < 0.05, **p < 0.01.

BODIPY-D-Alanine (7-BADA) and TAMRA-D-Alanine (5-TAMRA) probes were synthesized (Supplementary Fig. S5b) and found to be capable of effectively labeling bacteria in culture under anaerobic conditions for a duration of 48 h *in vitro* (Supplementary Fig. S5c). Calcium alginate gel was employed as a protective carrier for *in vivo* delivery of labeled bacteria into the gastrointestinal tract to maintain fluorescence signal retention (Supplementary Fig. S5d). Following oral gavage of bacteria (FDAA labeled) with the capsule coating, a diverse assortment of anaerobic bacteria, encompassing *B. uniformis*, *B. xylanisolvens*, *B. ovatus*, *P. copri* and *P. intermedia* were successfully imaged and monitored *in vivo* within live mice (Figure 4e). Importantly, *B. uniformis* showed a greater colonization stability in comparison to other bacteria, and could be found distributed throughout the entire intestinal tract (Figure 4e). This observation was also supported by *in situ* monitoring of the *B. uniformis* load for a duration of up to 21 days (Supplementary Fig. S6a).

Given the exceptional colonization capability of *B. uniformis*, we assessed the impact of *B. uniformis* on gut inflammation. Gavage of mice with live *B. uniformis* led to significantly reduced body weight loss induced by DSS relative to the medium control and heat-inactivated (dead) *B. uniformis* (Figure 4f, g). In addition, mice orally administered with live *B. uniformis* exhibited reduced colonic shortening and diminished histological indications of inflammation, as determined through a blinded assessment (Figure 4h–j). Subsequently, we conducted an analysis of intestinal immune cell populations in the mono-colonized mice through implementation of multi-parametric flow cytometry (Supplementary Fig. S6b). *B. uniformis* colonization significantly augmented the B cell population, whereas eliciting only modest impacts on other intestinal immune cells (Supplementary Fig. S6c). Similarly, the abundance of IgA-bound bacteria increased in mice treated with live *B. uniformis* (Figure 4k). To determine whether *B. uniformis* is involved in skewing *CD138*^+^ PC response toward IgA production, we analyzed the B cell lineage across peripheral blood mononuclear cells (PBMC), intestinal lamina propria mononuclear cells (LPMC) and the spleen (Supplementary Fig. S6d). Notably, *CD138*^+^ IgA^+^ PCs in both PBMC and LPMC exhibited a significant increase in *B. uniformis* group when compared to medium control and heat-inactivated groups (Figure 4l, m), while no increase was observed in spleen (Supplementary Fig. S6e). These findings suggest that *B. uniformis* have the potential to elicit the secretion of IgA by *CD138*^+^ PCs, which may subsequently coat gut bacteria to ameliorate DSS-induced inflammation.

### *Low IgA responses in* CD138*^+^ PCs fail in entrapping* B. uniformis *and aggravate colitis*

**Figure 5.**
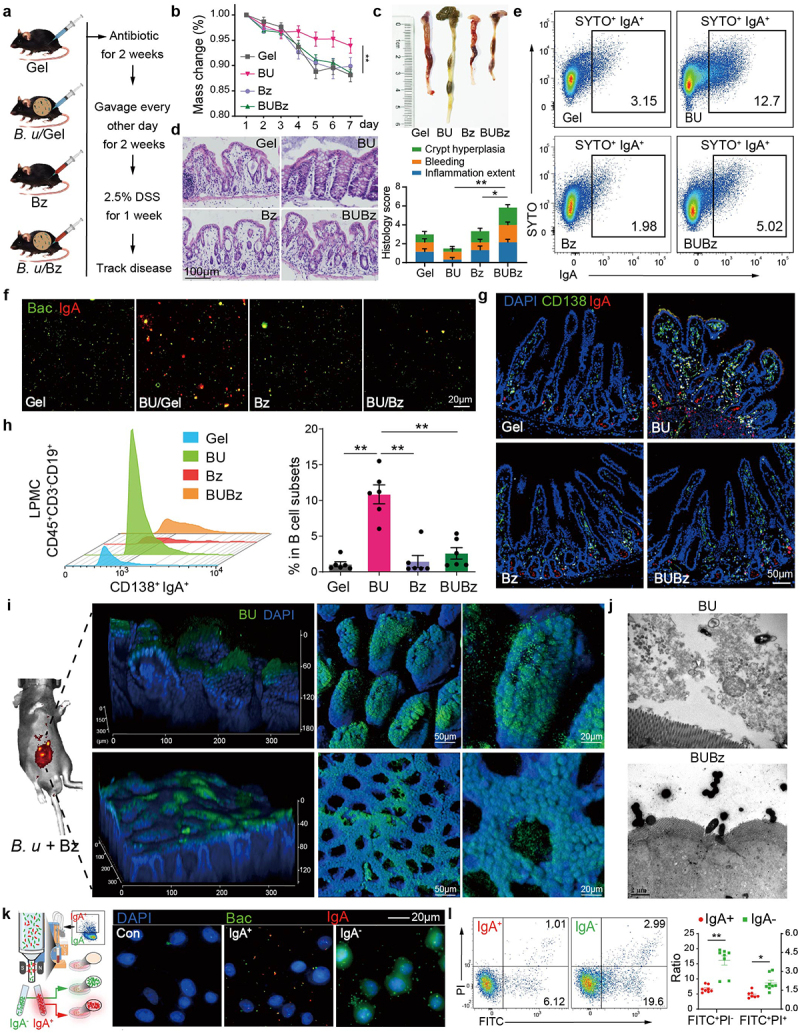
Low IgA responses in *CD138*^+^ PCs fail in entrapping *B. uniformis* and aggravate colitis.

We next asked what would happen if *CD138*^+^ PCs failed to produce IgA in response to *B. uniformis* in the gut. A mouse model with low IgA responses by administering a proteasome inhibitor bortezomib (Bz) via intraperitoneal injection was employed.^28^ First, we administered *Ruminococcus gnavus* ATCC29149 to Bz-treated mice, a strain known for inducing robust IgA responses in mice,^17,29^ to confirm the efficacy of Bz in dampening host-produced IgA responses (Supplementary Fig. S7a). Upon challenge with *Ruminococcus gnavus* ATCC29149, there was notable expansion of *CD138*^+^ PCs and vigorous IgA responses, accompanied by entrapment of bacteria in the gut (Supplementary Fig. S7b-g). Conversely, treatment with Bz effectively attenuated this cascade of reactions, confirming the reliability of the Bz-treated mouse model. Next, Bz-treated mice were colonized with *B. uniformis* for 2 weeks prior to DSS colitis induction (Figure 5a). Mice co-treated with *B. uniformis* and Bz exhibited exacerbated disease compared to those mono-colonized with *B. uniformis*, as documented by body weight, colon length, disease score, crypt hyperplasia and intestinal inflammation extent (Figure 5b–d). Furthermore, the increase of IgA-coated bacteria following *B. uniformis* gavage was abrogated in Bz-treated mice (Figure 5e, f and Supplementary Fig. S7h). Consistently, *B. uniformis*-mediated intestinal *CD138*^+^ PCs skewing toward IgA production was significantly attenuated by Bz (Figure 5g, h), concomitant with the development of severe gut inflammation. These findings suggest that *B. uniformis* sustains a symbiotic bond with the host by stimulating *CD138*^+^ PCs to produce IgA, whereas insufficient response from these effector cells would disrupt this association, ultimately exacerbating gut inflammation.

a, Mice were subjected for a 2-week regimen with gel (control), *B. uniformis*, Bz or *B. uniformis* + Bz followed by a one-week treatment with 2.5% DSS. b, Weight change of mice under DSS intervention for 7 days (*n* = 6). c, Macroscopic image of the colon on day 7 and length of colon. d, Representative micrographs of H&E-stained colonic sections. Comparison of the total histology scores and individual histological criteria scores across groups demonstrated that *B. uniformis* + Bz administration results in an increase in intestinal inflammation compared to *B. uniformis* group (scale bar, 100 µm; *n* = 6). e, Flow cytometry analysis of fecal bacterial IgA binding. f, Confocal images of gut luminal bacteria stained with SYTO BC (green) and anti-IgA-PE. Scale bars, 20 µm. g, Representative images of intestinal sections after IF staining with *CD138* (green) and IgA (red); scale bar, 50 µm. h, LPMC of all mice were collected for flow cytometry analysis of frequency of *CD138*^+^ IgA^+^ among live *CD45*^+^ *CD3*^−^ *CD19*^+^ cells, with quantification of *CD138*^+^ IgA^+^ precents (*n* = 6). i, 3D-reconstructed imaging of transplanted *B. uniformis* pre-labeled by 7-BADA in CUBIC-cleared ileum and colon tissue from *B. uniformis* + Bz treated mice. j, Representative TEM projections of intestine from mice subjected to *B. uniformis* and *B. uniformis* + Bz treatments (scale bar, 2 µm). k, Magnetically sorted IgA+ and IgA- bacteria from fecal samples and co-cultured with IPEC-J2 cells. Confocal images of immunofluorescence staining revealed a high level of cell adhesion in IgA-negative bacteria (scale bar, 20 µm). l, Magnetically sorted IgA^+^ and IgA^−^ bacteria from fecal samples and co-cultured with IPEC-J2 cells. Apoptosis was detected by concurrent staining with Annexin V-FITC and PI. Statistical analysis: (b) Two-way ANOVA, (d and h) One-way ANOVA or (l) unpaired *t*-test. *p < 0.05, **p < 0.01, mean ± SEM.

Next, CUBIC-based tissue clearing, *B. uniformis*-FDAA *in vivo* labeling, and whole-mount gut imaging protocols were utilized to enable 3D imaging of luminal *B. uniformis* (Supplementary Fig. S8a, b).^30,31^ Initially, little discernible *B. uniformis* labeled signal was detected in the gut luminal surface of mice subjected to *B. uniformis* gavage (Supplementary Fig. S8b). However, a remarkable bacterial fluorescence signal was detected in mice treated with a combination of *B. uniformis* and Bz (Figure 5i). Combined with our prior assessment of colonization ability of *B. uniformis*, we postulated that suppression of IgA response could facilitate the direct adhesion of *B. uniformis* to intestinal epithelium. Hence, using high-speed imaging mode,^32^ the biogeographic information of *B. uniformis* within intestine was acquired by scanning 65 ± 5 image planes from mucosal surface to the lamina propria, with a step size of 2.3 ± 0.7 μm between adjacent planes. As expected, low IgA response promoted *B. uniformis* attachment to the intestinal epithelium and formed discrete aggregates of densely packed cells atop the apical epithelium (Supplementary Fig. S8c). Ultrastructural imaging also revealed that in Bz-treated mice, *B. uniformis* penetrated the glycocalyx layer of transmembrane mucin, coming into close proximity with the microvilli (Figure 5j). To verify these optical imaging results, we performed qPCR comparing enrichment of *B. uniformis* across the lumen content, epithelial layer and mucosal tissue samples (Supplementary Fig. S8d). Consistent with prior findings, Bz treatment induced substantial translocation of *B. uniformis* from lumen to proximal mucosa after *B. uniformis* gavage, which was accompanied by an increased abundance ratio of epithelium-to-lumen in the Bz-treated samples (Supplementary Fig. S8e).

Given these results and that the observed spatial organization of *B. uniformis* was contingent on IgA-coating, we sought to investigate whether this physical interaction would affect steady-state epithelial cells. Bacteria from feces of mice treated with or without Bz that were isolated and applied to IPEC-J2 cell-line co-culture (Supplementary Fig. S8f). Fecal bacteria from the *B. uniformis* and Bz co-treated mice (low IgA response) significantly inhibited the viability of IPEC-J2 and promoted cell apoptosis (Supplementary Fig. S8f-h). To support the IgA-coated *B. uniformis* would affect steady-state epithelial cells, we employed IgA-based magnetic cell sorting to isolate IgA-coated and non-coated bacteria, those were then co-cultured with the IPEC-J2 cells, respectively. As expected, IgA-coated bacteria demonstrated a decrease in adhesion to epithelial cells (Figure 5k and Supplementary Fig. S8i), while non-coated bacteria caused reduced cell viability and promoted cell apoptosis (Figure 5l and Supplementary Fig. S8j). Together, these data converge to support that the lack of IgA secretion of *CD138*^+^ PCs leaded to the exacerbation of intestinal inflammation by *B. uniformis*, indicating the essential role of *CD138*^+^ PCs and IgA in the maintenance of *B. uniformis* commensalism.

### B. uniformis *exacerbates colitis in the absence of IgA or* CD138*^+^ PCs*

**Figure 6.**
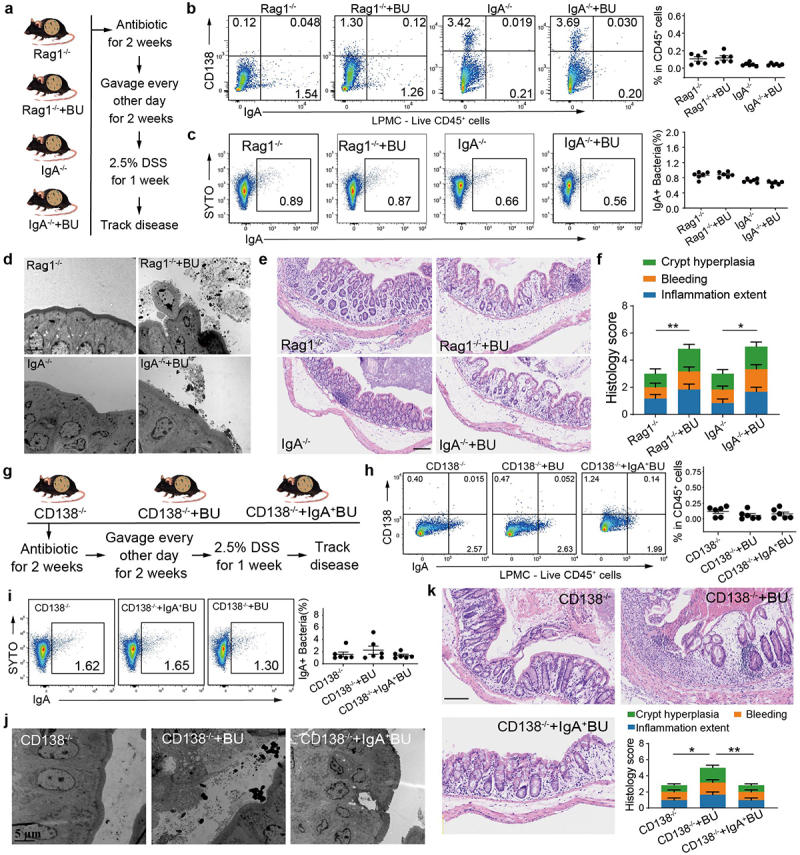
*B. uniformis* exacerbates colitis in the absence of IgA or *CD138*^+^ PCs.

To further elucidate the impact of IgA secretion by *CD138*^+^ PCs on the host-*B. uniformis* symbiosis and disease pathogenesis, IgA-deficient (*Rag*1^−/−^ or IgA^−/−^) mice were subjected to oral gavage with *B. uniformis* for 2 weeks (Figure 6a). Flow cytometric analysis determined a near absence of IgA and *CD138* double-positive B cells in the PBMC of the IgA-deficient mice (Supplementary Fig. S9a, b). Simultaneously, oral administration of *B. uniformis* to the IgA-deficient mice failed to elicit a *CD138*^+^ PC-skewed IgA response (Figure 6b). Both *Rag*1^−/−^ and IgA^−/−^ mice exhibited a remarkably depleted level of IgA-coated bacteria, with *B. uniformis* mono-colonized mice displaying extremely low levels (Figure 6c). Similarly, TEM images of colon tissues from mono-colonized IgA-deficient mice showed discrete aggregates of *B. uniformis* on the epithelial surface with direct contact to the microvilli (Figure 6d). As previously hypothesized, the disruption of the IgA-mediated bacterial agglutination reaction led to a compromised localization of *B. uniformis* to the mucosal linings, ultimately exacerbating inflammation. Specifically, administration of *B. uniformis* resulted in a reduction of colon length in IgA-deficient mice as compared to the control group (Supplementary Fig. S9c), and was accompanied by the onset of severe intestinal inflammation characterized as crypt distortion and mucosal ulceration (Figure 6e, f).

a, *Rag*1^−/−^ and IgA^−/−^ mice were subjected for a two-week regimen with medium (control) or *B. uniformis*, followed by a one-week treatment with 2.5% DSS. b, LPMCs of *Rag*1^−/−^ and IgA^−/−^ mice were collected for flow cytometry analysis of frequency of *CD138*^+^ IgA^+^ among live *CD45*^+^ *CD3*^−^ *CD19*^+^ cells, with quantification of *CD138*^+^ IgA^+^ precent (*n* = 6). c, Flow-cytometry analysis of fecal bacterial IgA binding across groups, with quantification of the IgA-bound fraction (*n* = 6). d, Representative TEM projections of intestine from *Rag*1^−/−^ and IgA^−/−^ mice subjected to control or *B. uniformis* treatments (scale bar, 2 µm). e, Representative micrographs of H&E-stained colonic sections, demonstrating that *B. uniformis* causes an increase in intestinal inflammation relative to controls (scale bar, 100 µm). f, Comparison of the total histology scores and individual histological criteria scores across groups (*n* = 6). g, *CD138*^−/−^ mice were gavaged for 2 weeks with medium (control), *B. uniformis* or magnetically sorted IgA-positive *B. uniformis* and treated with 2.5% DSS for 1 week. h, LPMCs of *CD138*^−/−^ mice were collected for flow cytometry analysis of frequency of *CD138*^+^ IgA^+^ among live *CD45*^+^ *CD3*^−^ *CD19*^+^ cells, with quantification of *CD138*^+^ IgA^+^ precents (*n* = 6). i, Flow cytometry analysis of fecal bacterial IgA binding across groups, with quantification of the IgA-bound fraction (*n* = 6). j, Representative TEM projections of intestine from *CD138*^−/−^ mice treated with medium (control), *B. uniformis* or magnetically sorted IgA positive *B. uniformis* (scale bar, 2 µm). k, Representative micrographs of H&E-stained colonic sections. Comparison of the total histology scores and individual histological criteria scores across groups demonstrate that *B. uniformis* causes an increase in intestinal inflammation of *CD138*^−/−^ mice relative to other groups (scale bar, 100 µm; *n* = 6). Statistical analysis: (b, c, h, i and k) One-way ANOVA. *p < 0.05, **p < 0.01. ns, not significant. Mean ± SEM.

We next employed a *CD138*^−/−^ mouse model to elucidate the specific effector function of *CD138*^+^ PC. We performed gavage colonization assays using IgA-coated *B. uniformis* (sorted magnetically) alongside wild-type *B. uniformis* (Figure 6g). After 2 weeks colonization, low levels of IgA^+^ cells were detected in LPMC samples from all experimental groups, although a small amount of IgA secretion persists in the intestine of *CD138*^−/−^ mice (Figure 6h). Notably, in both free and IgA-coated *B. uniformis* gavaged mice, the abundance of IgA-coated bacteria remained consistently low and comparable to that of the control group (Figure 6i). These results confirmed that the high levels of IgA-coated bacteria in *B. uniformis*-treated mice were predominantly driven by *CD138*^+^ PC-derived IgA. Moreover, in *CD138*^−/−^ mice subjected to *B. uniformis* gavage, the characteristic aggregation of epithelium was observed as a consequence of the weakened agglutination response by IgA (Figure 6j). Conversely, no such effect was observed in mice treated with IgA-coated *B. uniformis*. We subsequently assessed inflammatory score data. Remarkably, significantly elevated colitis and crypt hyperplasia scores were observed in the group treated with free *B. uniformis* compared to the control and IgA-coated *B. uniformis* treated groups (Figure 6k), suggesting that IgA coating prevents the pro-inflammatory effects of *B. uniformis*. Together, these results suggest that *B. uniformis* can exacerbate colitis unless coated by IgA produced by *CD138*^+^ PCs.

### CD138*^+^ PCs mediate interactions with enterocytes and bacterium in weaning stress*

**Figure 7.**
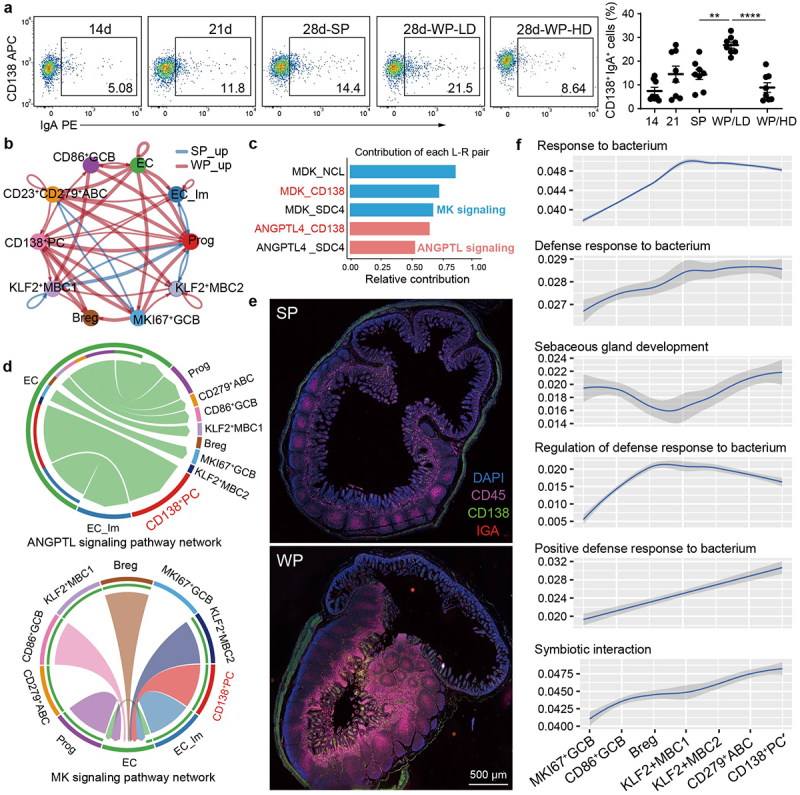
*CD138*^+^ PCs mediate interactions with enterocytes and bacterium in weaning stress.

Finally, we explored whether weaning stress deepened the connections of *CD138*^+^ PCs, intestinal epithelial cells and gut bacteria in piglets. To profile the ontogeny and expansion of *CD138*^+^ PCs within the intestinal milieu, flow cytometry was utilized to analyze the temporal alterations in the dynamics of *CD138*^+^ PCs during the pre- and post-weaning periods. *CD138*^+^ PCs were observed to be present in piglets prior to weaning and their proportions further elevated with age. Notably, in the WP group, the frequency of IgA-secreting *CD138*^+^ PCs exhibited a discernible contrast between the low diarrheal rate (LD) cohort, where it was notably elevated, and the high diarrheal rate (HD) cohort, where it was conspicuously diminished (Figure 7a). The aggregated cell–cell communication network revealed that piglets with weaning stress had higher numbers of interactions and stronger interaction strengths (weights) (Supplementary Fig. S10a, b). Especially, there was a stronger interaction between *CD138*^+^ PCs and various types of epithelial cells in the WP group than the SP group (Figure 7b and Supplementary Fig. S10c). To rank the significant pathways, the differences in overall information flow was evaluated within the inferred networks. Our findings indicate that the MK and ANGPTL signaling pathways exhibited a higher degree of enrichment in the WP group compared to the SP group (Supplementary Fig. S10d). Next, we extracted all the ligand-receptor pairs with significant contribution and related signaling genes for the two given signaling pathways (Figure 7c). Not only did the ligand-receptor pairs of *MDK-CD138* and *ANGPTL4-CD138* contribute significantly to the signaling pathways, but also they played a crucial role in mediating interactions between enterocytes and *CD138*^+^ PCs (Figure 7d). To verify the interaction relationship inferred from *in silico* analysis, we conducted multiplexed immunofluorescence (IF) labeling for *CD45*, *CD138*, and IgA in intestinal tissue sections. In the inflamed intestinal tissue of the WP group, we observed notable recruitment of *CD138*^+^ IgA^+^ PCs, which extensively infiltrated the lamina propria and intestinal epithelium (Figure 7e). In addition to cell–cell interactions, we collected and curated 27 signaling pathways relevant to gut bacteria related response, and scored all B cell subsets accordingly. By comparing the differences in pathway enrichment among the populations, we identified 14 pathways distinctly enriched in the *CD138*^+^ PC population, with higher levels of enrichment compared to other B cell subsets (Figure 7f and Supplementary Fig. S10e). Collectively, these findings provide evidence for the link between *CD138*^+^ PCs, epithelial cells and bacteria in piglets experiencing the weaning stress.

a, Flow cytometry analysis of frequency of *CD138*^+^ IgA^+^ PCs in piglets collected at days 14, 21 (per-weaning) and 28 (post-weaning) with quantification of the *CD138*^+^ IgA^+^ PC subset across groups (*n* = 8). One way-ANOVA, **p < 0.01, ****p < 0.0001, mean ± SEM. WP/HD: weaning piglets with high diarrheal rate, WP/LD: weaning piglets with low diarrheal rate. b, Circle plot showing differential cell–cell communication network between two groups. The width of edges represents the relative number of interaction strength. Red colored edges represent increased signaling in the WP group. c, Histogram showing significant interactions (ligand-receptor pairs) and related signaling genes for the given signaling pathways. d, Chord diagram visualizing the cell–cell interactions mediated by two selected signaling pathways. e, Representative images of intestine sections from SP and WP group after IF staining with *CD45* (pink), *CD138* (green) and IgA (red); scale bar, 500 µm. f, Individual cell AUC score for host–microbiota interaction activities were subjected to statistical analysis, followed by liner regression analysis with the ranking of each B cell cluster.

## Discussion

In the early stages after birth, the gut microbiota of mammals is first shaped by the dietary and immunological components of milk and then changed by the introduction of solid food upon weaning.^33^ At this period, the developing gut microbiota elicits a robust immune response – a critical event called “weaning reaction” – which serves to safeguard the infant against pathogenic microorganisms that at the same time promote tolerance to commensal bacteria.^3,34^ The emergence and maintenance of immune-microbe relationships during the weaning period have gained much attention. While many investigations have bettered our understanding of the weaning process,^35^ the precise mechanisms remain unclear on how immune-microbe mutualism and the perturbation of such affect intestinal homeostasis following weaning-induced microbial expansion. Piglets represent a promising animal model for exploring the immune education elicited by the microbiota during the weaning period.^36^ The piglet model bears striking resemblance to the pathophysiology of microbe-associated gut disorders in infants, providing a powerful tool that does not subject to the constraints of human clinical studies.^19,37^ Our findings using the piglet model validated that the weaning stress has a deleterious impact on growth performance, intestinal morphology and barrier function. It provides a “window of opportunity” for the disruption of the symbiotic relationship between gut microbiota and the host, leading to the reconstruction of immune microenvironment. The present study focuses on the interplay between gut microbiota communities and the finely orchestrated adaptive immunity mediated by B cells. We found that the weaning stress induced substantial infiltration of B cells during the onset of intestinal inflammation. Intriguingly, the gut bacterial communities changed in weaning piglets with increased proportions of bacteria coated with IgA, consistent with the results of previous studies.^35,38^ However, these findings raised more questions than answers. What is the mechanism underlying the dynamics of microbial community shifts during the weaning process? Why are B cells and IgA there? Are there specific microbial taxa mediating the observed effects?

Weaning can indeed expedite the temporal evolution of gut microbiota in a fierce manner. This phenomenon likely arises from the deterministic filtering effect of weaning stress,^19^ which favors the proliferation of rapidly multiplying bacteria while tempering the growth of slower-growing counterparts, thus triggering a cascade of dynamic shifts in diverse microbial taxa. In that, Bacteroidetes taxa displayed a heightened reactivity to the weaning process. As demonstrated in previous studies,^16,39^ weaning fosters the proliferation of anaerobes, specifically the Bacteroidetes phylum, wherein the Bacteroides genera make up the predominant members in our investigation. Interestingly, our findings revealed a moderate correlation between the abundance of Bacteroides and the incidence of weaning-associated diarrhea in piglets, which deviated from previous reports that displayed a significant association.^40^ To clarify, in a specific subset of the weaned cohort, an increase in Bacteroides abundance and concurrent reduction in diarrhea incidence were observed in some individuals. Here, we took the IgA-coated bacteria into the consideration. Piglets were reconfigured into high or low groups according to the levels of IgA-coated bacteria. Strikingly, within the group of low IgA-coated bacteria, the prevalence of Bacteroides exhibits a stronger correlation with the index of diarrheal symptoms compared to the highly coated counterpart. Thus, it is hypothesized that the commensal species leverages IgA to promote colonization in the gut, while the compromised IgA response led to dysbiosis of microbial community and increased susceptibility to gastrointestinal infections.^11,12^ To ascertain the unique capacity of individual bacterial species to induce IgA, the high abundance bacteria in the WP group, such as *B. uniformis*, *B. xylanisolvens* and *B. ovatus* were isolated and cultured. The isolated bacteria were discerned in discrete colonization trajectories through sequencing and alignment.

With single-cell transcriptomic sequencing analysis, a divergence was identified in the developmental trajectory of intestinal epithelial cells between the breastfeeding and weaning. Specifically, while a reported developmental trajectory existed at the early stages of development,^41,42^ an alternative gene expression program was identified to be executed by a subset of mature enterocytes in the WP group. Weaning stress elicits a pronounced enrichment of signaling pathways related to B cell interactions within the enterocyte population. In the WP group, the landscape of infiltrating B cells was characterized by *CD138*^+^ PCs. Simultaneously, *CD138*^+^ PCs from the WP group exhibited an abnormal shift toward a specific IgA-producer cluster, which was verified by flow cytometry and IF analyses. Previous studies on inflammatory bowel disease have reported a PC-skewed humoral response, indicating that dysregulation of IgA secretion may impede the ability to modulate particular inflammatory commensals, thus initiating intestinal inflammation.^26,27^ Geneset signature analysis provided further evidence that *CD138*^+^ PCs may mediate the maintenance of host-gut microbiota symbiosis.

Next, we investigated whether *CD138*^+^ PCs were the effector cells monitoring the commensal bacteria to inhabit in the gut of young animals to ensure the preservation of steady-state conditions. Certainly, functional differences at the species level are crucial in determining the protective or disease-enhancing properties of the commensal bacteria and represent a fundamental factor that impacts immune-microbe interactions during early life.^1,17^ In terms of colonizing abilities and differences in IgA-inducing capacity, *B. uniformis* as an isolated species had the highest capacity for inducing gut IgA production than other isolates, as well as enhancing the proportion of the IgA-secreting *CD138*^+^ PCs. The activation of *CD138*^+^ PCs resulted in high abundance of IgA-coated bacteria and attenuation of DSS-induced colitis in *B. uniformis* mono-colonized mice. Remarkably, depletion of IgA response of *CD138*^+^ PCs decreased *B. uniformis* entrapment adjacent to intestinal mucosa and reshape the composition and topography of *B. uniformis* in gastrointestinal tract. This result is in line with the concept that the immune system selectively recognizes commensals through sensing pathogen-associated activities such as adherence to the intestinal epithelium, ability to colonize sterile mucosa, or invasion into tissue.^9,43^ Indeed, we next revealed that IgA coating weakened *B. uniformis* to adhere to enterocytes and had an apoptosis-promoting property *in vitro*. Previous studies have suggested that IgA response tempers the expansion of the constitutive inhabitants, particularly those with inflammatory potential, and inhibits inflammation caused by bacterial invasion.^44^ As such, unconstrained expansion of particular symbiotic bacteria occurs in the absence of an efficacious IgA response.^45^ Furthermore, IgA targeting these bacteria mitigates intestinal inflammation by means of bacterial exclusion.^46^ As a limitation, regarding the specificity of the IgA response, our study focused primarily on elucidating the role of *B. uniformis* in inducing IgA responses within *CD138*^+^ PC and its influence on host-microbial symbiosis during the weaning period. The specific versus nonspecific nature of IgA response to *B. uniformis* or other gut bacteria warrants further investigation. The colonization assays in knockout mice indicated that the deficiency in *CD138*^+^ PCs responded to the *B. uniformis* population to disrupt a state of non-inflammatory commensalism where the IgA-coated bacteria were maintained at low levels. These behaviors suggest the quiescence of *CD138*^+^ PCs during the weaning process, when *B. uniformis* expansion has an impact on inflammatory responses and exacerbated disease susceptibility. We acknowledge all our mechanistic investigations were conducted in colitis models of adult mice. Future studies will continue to seek direct correlations under weaning stress models. To address this limitation, we finally transitioned our focus back to piglet experiments examining the cessation of breastfeeding. A remarkably intimate interaction was revealed between the host and commensal bacteria during the weaning process, which was effectively facilitated by *CD138*^+^ PCs. However, this interaction turned into a pro-inflammatory phenotype and thereby instigated intestinal inflammation in the event of *CD138*^+^ PCs dysfunction.

In conclusion, the symbiotic interaction between *B. uniformis* and the young mammalian host is facilitated by a provocation of *CD138*^+^ PC response that skewed toward the IgA production, which enables crosslinking of luminal bacteria. Otherwise, insufficient IgA-mediated agglutination from *CD138*^+^ PC may lead to an uncontrolled expansion and altered topography of *B. uniformis*, promoting its encroachment on the epithelial cells and aggravating gut inflammation induced by the weaning reaction. These findings enhance our understanding of intestinal inflammatory pathologies during the weaning transition and hold implications for interventions.

## Materials and methods

### Animals and cell culture

All animal experimental procedures were permitted by the Zhejiang University Institutional Animal Care and Use Committee under the permission code ZJU20210301. A total of 72 piglets born from Landrace × Yorkshire sows were used, with 36 WPs and 36 SPs. In the WP group, piglets were weaned at 21 days of age then given creep feeds. Piglets in the SP group stayed with the nursing sows. Growth performance and diarrhea index of each piglet from 21 to 35 days of age were recorded. Rectal swab samples were harvested at lactation phase (7, 14, and 21 days) and weaned phase (28 and 35 days) for longitudinal microbiome analysis. Serum, feces, and entire segments of ileum and colon tissues containing lumen content and mucosae were collected. Pathological scores of intestinal H&E stains were read in a blinded manner and quantified.

Specific pathogen-free (SPF) C57BL/6 mice (male, six-week-old, weight 15 ± 2 g) were obtained from the Shanghai Model Organisms Center. SPF *Rag1*^−/−^ mice (male, five-week-old, weight 12 ± 2 g) were imported from the Jackson Laboratory. B6/ JGpt-*Igha*^em1Cd7691^/Gpt (*Igha*-KO) and B6/JGpt-*Sdc1*^em22Cd8408^/Gpt (*CD138*-KO) mice were purchased from GemPharmatech. All mice studies were carried out under SPF conditions with a 12-h light/dark cycle. Mice were housed in individually ventilated cages under strict barrier conditions with free access to autoclaved diet and water.

The cell-line IPEC-J2 was cultured in DMEM/F12 medium (Gibco) supplemented with 10% fetal bovine serum (FBS, PD Pharmingen) and 1% Penicillin-Streptomycin (Pen-Strep, Gibco). Cells were grown in a Model 6856 Incubator (Thermo Scientific) with 95% humidity and 5% CO_2_ at 37°C.

### Adhesion and invasion assay

The adhesion and invasion assay were performed using IPEC-J2 cells exposed to fecal bacteria from *B. uniformis* colonized mice, IgA-coated bacteria, or isolated *B. uniformis* according to a published procedure.^47^ Briefly, cell suspension (500 µl) without Pen-Strep was seeded onto 20 mm round coverslips placed in the wells of a 12-well plate with a cell density of 10^6^ cells per well. After growing to 80% confluency, live bacteria samples were dropwise added into plates (5 × 10^6^ bacteria per well). At 2, 4, and 6 h post-innoculation, IPEC-J2 cells were collected for subsequent CCK8, apoptosis detection, and immunofluorescence (IF) assays.

### Bacterial strains

For *Bacteroides* species isolation, genomic DNA of feces derived from weaned piglets and *Bacteroides*-specific primers were used for a qPCR screen. The feces with cycle threshold <18 for the primers were selected as *Bacteroides*-positive feces. *Bacteroides*-positive feces were homogenized in thioglycollate broth medium (LA8740, Solarbio), and then anaerobically incubated on deoxidized Schaedler agar plates (DSMZ Medium 1669) using quadrant streaking method. The plates were placed in a vinyl anaerobic chamber (Coy Drive 14,500, USA) and anaerobically cultured at 37°C for 36 h. The resulting colonies were isolated and screened based on the DNA sequence of the 16S rRNA region using the Sanger sequencing workflow, after which the sequences were matched to known *Bacteroides* spp. using NCBI Nucleotide BLAST (megablast >99% of similar sequences). Bacteria strains *Ruminococcus gnavus* ATCC29149, *Prevotella copri* DSM 18,205 and *Prevotella intermedia* DSM 20,706 were obtained from the American Type Culture Collection (ATCC) or German Collection of Microorganisms and Cell Cultures (DSMZ), as indicated by the strain names. *Bacteroides uniformis*, *Bacteroides xylanisolvens* and *Bacteroides ovatus* were isolated from feces of weaned piglets as described above. Brucella Blood Agar (BRU, Anaerobe Systems), Laked Brucella Blood Agar with kanamycin and vancomycin (LKV, Anaerobe Systems), BRU/LKV (Anaerobe Systems) biplates were used for *Proteus mirabilis*, *Fusobacterium varium*, *Prevotella copri* and *Prevotella intermedia* isolation, respectively. All bacteria were stored in glycerol at −80°C for long-time perseveration.

### Oral gavage and induction of colitis in mice

Mice were randomly housed in pairs and treated with three stages of antibiotics (see below).^48^ They were sequentially gavaged with 100 µl calcium carbonate and 10^8^ bacteria diluted in 200 µl PBS, with heat-inactivated bacteria as the negative control and culture medium as the blank control. Oral gavage was performed every 3 days for 21 days. Colitis was induced by administration of dextran sodium sulfate (DSS, 2.5% in drinking water, MP biomedicals) from days 14 to 21 after bacterial gavage. Body weight and bacterial load were monitored until mice were sacrificed. Whole blood, spleen, ileum, colon, and lumen content samples were collected.

### Three-stage antibiotic treating regime

Stage 1: Antibiotic cocktail containing ertapenem sodium, neomycin sulfate, and vancomycin hydrochloride was added into drinking water for 3 days. Stage 2: Antibiotic cocktail containing ampicillin, cefoperazone sodium salt, and clindamycin hydrochloride was added into drinking water for 3 days. Stage 3: Repeat of Stage 1 followed by clean water for 2 days prior to gavage. Then, the antibiotic-free diet and drinking were provided during the entire bacterial strain colonization trial. Antibiotics were from MedChemExpress or Sigma. The dose of each antibiotic was 1 mg/ml in drinking water.

### Intestinal plasmacyte depletion

Stock solution of Bortezomib (Bz), a proteasome inhibitor,^28^ was prepared by dissolving 5 mg Bz (PS-341, MedChemExpress) into 200 µl DMSO under ultrasonic agitation. Carboxymethylcellulose sodium (10 mg/ml in distilled water, C8621, Solarbio) was mixed with a diluted Bz solution (60 µg/ml in PBS) to formulate a hydrogel. A volume of 200 µl hydrogel containing Bz, or control hydrogel without Bz, was drawn into a sterile syringe and administered to seven-week-old mice by intraperitoneal injection. Two days after that, mice received a secondary intraperitoneal injection. Twelve hours later, the degree of plasmacyte depletion was assessed by ELISA, IF and flow cytometric assays. When the depletion was achieved, sequential oral gavage was performed in the plasmacyte-depleted mice, along with additional hydrogel administration.

### Intravital imaging

For whole-body intravital imaging of fluorescent *B. uniformis*, mice underwent general anesthesia with vaporized isoflurane (2.5% liter/min) provided in air anesthesia ports. Optical overlays of fluorescence signals on photographic images of mice were acquired with the IVIS imaging platform (IVIS Lumina XRMS system, PerkinElmer). Workflows for imaging acquisition and analysis implemented in the software imaging wizard (Living Image Software, PerkinElmer) followed three steps: setting excitation and emission filters for 570 and 520 nm, respectively; adjusting Field of View (FOV)-E; and manually setting exposure time to 5 s. For *Ex vivo* imaging, intestines dissected from mice were immediately transferred into PBS at 4°C and gently trimmed of fat tissue, mesentery lymph nodes, and blood from the intestinal surface. The intestines were then placed into the imaging chamber with FOV-D setting adjustment. Luminescent exposure time was set to 1.2 s. All other setting parameters were left unchanged.

### Imaging of B. uniformis in the gut

Fluorescent D-amino acids labeling strategy was adopted for in-depth delineation of *B. uniformis*-host interaction.^30,49^ Briefly, *B. uniformis* was cultured in the vinyl anaerobic chamber at 37°C. 7-BADA (0.75 mM) or 5-TAMRA (0.5 mM) were blended within the culture medium when the bacteria reached the log-phase, then co-inoculated overnight. The fluorescent signal of the labeled bacteria was detected every 8 h *in vitro* and exhibited reliable labeling property for up to 48 h. To avoid decay of the fluorescence intensity within intestinal tissues, calcium alginate gel was used as a protective vehicle for delivering the labeled bacteria. Sodium alginate (2.5% w/v), cryoprotectant (3% w/v whey protein, 15% v/v glycerin and 4% v/v mannitol) and the labeled bacteria suspension (approximately 10^9−^10^10^ CFU/µl) were mixed evenly, generating capsules in 1 M calcium chloride solution by hardening for 30 min. Mice received 200 mg of the capsules twice via oral gavage with a 6-h interval. After that the mice were euthanized, and the intestinal tissues were fixed for 1 h in 4% paraformaldehyde for CUBIC processing. We performed tissue clearing using modified CUBIC-reagent-1A containing Triton X-100 (15% w/v), urea (25% w/v), quadrol (20% w/v) and distilled water. After dissolution, the reagent was added NaCl to 20 mM final concentration. The fixed tissues were washed 3 times with PBS to remove paraformaldehyde, and immediately incubated in half-diluted CUBIC-reagent-1A for 4 h at 4°C with shaking (60 rpm). Then, the tissues were placed into CUBIC-reagent-1A (not diluted) for 2–3 d at 4°C with shaking (40 rpm), and exchanged the fresh reagent with 1 d interval. The tissues were cut into 4 × 4 mm blocks when they were sufficiently transparent, after which the tissues blocks were stained with DAPI (10 µg/ml) in PBS for 20 min with no shaking. The samples were gently washed with PBS to discard the nuclear dye, immobilized onto slides with tissue-adhesive glue and dripped mounting medium onto the sample for imaging application. Confocal Z-Stack imaging was captured on an inverted confocal laser scanning microscope (Zeiss LSM 880, Germany) using objective plan- apochromat 10× eye piece and 60× oil lens. Acquisition parameters of Z-Stack procedure were setting on ZEN 2012 software, including fluorescence channels, the first and last focus area location, scanning through to the bottom and adjusting scan slice interval. Original images and documents were submitted to ZEN 2012 software for post-analysis and video editing.

### DNA purification and qPCR assay

For DNA extraction, samples were harvested from isolated bacterial cells, intestinal lumen content, epithelial layer and mucosal tissue following previously described guides with modifications.^50^ Briefly, isolated bacteria were pelleted and quickly processed to DNA extraction using SteadyPure Bacteria Genomic DNA Extraction Kit (Accurate Biotechnology). For intestinal lumen content, freshly dissected colon segment was opened longitudinally. Lumen content was sampled, vortexed, and weighted. The remaining tissue was gently rinsed with sterile Hank’s Balanced Salt Solution (HBSS, Invitrogen) to remove residual lumen content. Mucus layer was scrapped from the mucous membrane of the washed tissue using a sterile coverslip and collected. Tissue homogenates from 100 mg of the remaining tissue were generated in 1 ml PBS using an Ultra-Turrax homogenizer. Specific primer pairs of V3-V4 region of *B. uniformis* 16S rRNA gene were designed and prepared to PCR reaction mixtures according to the protocol of SYBR Master Mix (Roche). Genomic DNA from isolated *B. uniformis* was used to graph a qPCR standard curve, which allowed to detect the absolute quantity of *B. uniformis* in all samples.

### 16S rDNA sequencing

Well-mixed fecal from each animal sample was collected to extract microbial DNA using the CTAB method. Negative control (nuclear-free water) was included. The region V3-V4 of 16S rDNA genes were PCR amplified following pervious method.^51^ The PCR products were cleaned by AMPure beads (Agencourt), quantified using Qubit assay kit (Invitrogen) and then prepared in equimolar concentrations. The amplicon libraries were submitted for Illumina NovaSeq PE250 platform to generate 2 × 250 bp paired-end reads. Next, the sequencing output was processed with QIIME2 pipeline. After assigning to samples, primer and barcode trimming, merging paired-end reads, and quality score-based filtering. The clean tags were dereplicated using DADA2 and subsequently obtained feature (amplicon sequence variant, ASV) sequence and table. The ASV sequences were taxonomically classified with SILVA reference database (Version release 138). Alpha and beta diversity were evaluated according to the ASV table.

We calculated Pearson correlations of genus abundance matrix across time- and treatment-series condition, followed by modeling microbial co-occurrence ecological networks, which were carried out in R (igraph package). Indices of taxon mean proportion and detection ratio of discrete samples were calculated and ranked on the basis of genus abundance matrix. Curves of Occupancy-abundance distribution were plotted using an established workflow,^52^ which enable to directly inform core microbiome species in our designs. Sorting and visualizing the mean relative abundance of each individual taxa were implemented with the ggalluvial package. We carried out linear discriminant analysis using the lda function in MASS v.7.3–57 package to differentiate between microbial community in each sampling point.

### Cell suspension and library preparation for single-cell RNA sequencing

The ileum tissue was carefully sliced into fragments of approximately 0.25 mm^2^ and immersed in a digestion solution containing 2.5 mg/ml papain, 0.45% collagenase IV, and 180 units/ml DNase I, and digested at 37°C for 15 minutes. To halt enzymatic activity, 20% (v/v) FBS was added, followed by gentle pipetting for six repetitions. The cell suspension obtained after passing through an 80-μm strainer was centrifuged at 500 × g for 5 minutes at 4°C. The resulting pellets were mixed with resuspension buffer (0.04% FBS) and red blood cell (RBC) lysis buffer, followed by incubation at 37°C for 5 minutes. Damaged cells were removed using a Miltenyi Dead Cell Removal Kit (MACS, 10× Genomics). Total cell counts ranging from 900 to 1200 cells/μl and viability (above 90%) were determined using a hemocytometer/Countess II Automated Cell Counter (Thermo Fisher). Subsequently, the cell suspension was processed using the 10× Chromium single-cell platform, following the manufacturer’s protocol for the Chromium Single Cell 3’ V3 assay (10× Genomics, USA). The cDNA amplification and library construction were performed following the manufacturer’s recommendations. Finally, the libraries were subjected to paired-end multiplexing sequencing on the Illumina NovaSeq 6000 platform, generating 150 bp reads.

### Single-cell RNA sequencing analysis

Raw FASTQ files were trimmed to remove the marker adapters and imported into Cellranger v.3.0 pipeline to preform cellular barcode demultiplexing, reads alignment (pig reference genome, *sus scrofa* v96), UMI quantification, and read annotation. Subsequently, raw UMI count matrix and the corresponding gene information were acquired. We performed Seurat object generating, data filtering and quality control using Seurat v.4.1.1 R package, where cells showing fewer than 500 or more than 4500 feature genes, and those with 10% mitochondrial gene content detected were excluded in downstream analysis.

After selecting top variable features, setting covariate parameters, scaling and centering data, we initially ran a principal component analysis (PCA) and computed *p* value for each pinicipal component (PC) using RunPCA and JackStraw function. The top 20 significant PCs were input as dim parameters and the k. param-nearest neighbor algorithm was employed for the dataset. UMAP algorithm was performed to plot cell clusters with resolution set to 0.5. Marker genes of cell clusters were detected using a Wilcoxon rank sum test via FindAllMarkers function in Seurat wrapper and visualized using dot or violin plots. Two major B cell clusters (highly expressed *CD79A*, *JCHAIN*, *CD19* and *CD20*) were extracted and integrated into a new object. Then, PCA, estimating significant dimensions, clustering analysis and UMAP re-embeddings were performed as mentioned above.

### Cellular pseudotime trajectories

Cellular pseudotime trajectories were constructed using Monocle3 v1.2.9 algorithm. Briefly, epithelial cells and mature enterocytes were separately extracted from the integrated Seurat object using subset function and created into new cell data set (CDS) objects using new_cell_data_set function in Monocle3 package. The CDS objects were pre-processed, reduced dimensionality and visualized in UMAP according to recommended analysis procedures. All cells were assigned and clustered to different partition after calling cluster_cells command with default parameters. Principal graph was fitted within partitions by running learn_graph function. For the epithelial cell CDS, progenitor cell region was manually defined as “root” spot of the pseudotime graph using order_cells function and the resulting graph was fully connected from progenitor cell to mature enterocytes. For the enterocyte CDS, we observed a branch node in which cells from weaned or suckling groups traveled to distinct cellular “decisions”. Additionally, we carried out trajectory inference using Monocle v.2.20.0 package to reconfirm the above predicted process and obtained similar results. Based on that, grouping differential genes that co-varied across pseudotemporal expression pattern were performed using plot_pseudotime_heatmap function. For branch in enterocyte trajectory, plot_genes_branched_heatmap command was run with default parameters to inspect branch-dependent gene expression patterns.

### AUCell

To analyze whether single cells were enriched with the loaded gene-sets, we applied AUCell v.1.14.0 package and ran standard commands as previously described.^53^ Individual expression matrix profiles of enterocyte or *CD138*^+^ B cell clusters were subset to generate the recommended sparse format. The canonical gene-sets database was obtained from MSigDB CP: KEGG and C5: GO gene-sets. B lymphocyte-related signaling pathways were used in downstream analyses including “B cell receptor signaling pathway” and “Intestinal immune network for IgA production”, and 24 responses to bacterial-related signal pathways were sorted out from MSigDB C5: GO database. Gene-set enrichment scores were calculated by running AUCell_buildRankings and AUCell_calcAUC commands, and the resulting AUC scores were returned into the matrices. Data frame was extracted containing single-cell UMAP embedding parameters and AUC scores for each cell, and input into ggplot2 v.3.3.5 package for visualization. For B cell subpopulations, the AUC score histogram of IgA production gene-set was plotted by running AUCell_exploreThresholds command. We set a threshold to define IgA production highly “active” cells, and found that most *CD138*^+^ B cells were assigned above the given threshold. Functional pathway enrichment analysis of enterocyte-related differentially expressed genes between groups was implemented in GSEABase v.1.54.0 package.

### CellChat

To delineate atlases of intercellular communication between epithelial cell and B cell subsets, CellChat v.1.4.0 package was employed to infer cell–cell interaction network and identify dominant contributing signals to communication patterns. Specifically, B cell and epithelia cell subsets were isolated from initial Seurat object and built to generate two CellChat objects according to grouping information using createCellChat function. CellChatDB database was loaded as standard ligand-receptor pairs database. Next, by default, standard predicted procedure of CellChat analysis was performed with the following steps: 1) applying “trimean” algorithm in computeCommunProb function for obtaining ligand-receptor pair numeric counts; 2) computeCommunProbPathway function for computing signaling pathway levels; 3) aggregateNet function for calculating the aggregated networks. Then using mergeCellChat function integrated CellChat objects of two groups and compared number or strength of interactions between two groups by setting measure = “count” or “weight”. Mean ranking signaling network comparison for two groups was implemented in rankNet function. The signaling pathway and specific ligand-receptor gene pairs contributing to cell–cell communication were visualized by chord diagram and histogram.

### Cell suspension preparations

RBC lysis (Biosharp) was performed twice to obtain peripheral blood mononuclear cells (PBMCs). Splenocytes were separated by mincing spleen tissues, filtering through a 70-µm cell strainer, and RBC lysis. Lamina propria mononuclear cells (LPMCs) were obtained following the removal of lumen contents, fat and mucosal layer from intestinal tissues. The remaining tissues were cut into pieces, transferred into HBSS containing 5 mM EDTA and 1 mM DTT, and shaken for 20 min at 37°C. Tissues were washed using complete RPMI medium (RPMI-1640, Gibco, containing 10% FBS and 1% Pen-Strep) then incubated in enzymatic digestion buffer (all reagents from Sigma Aldrich) containing collagenase D (1 mg/ml), Dispase (7.5 U/ml) and DNase (5 U/ml) for 30 min at 37°C with shaking. The digested cells were allowed to pass through a 70-µm cell strainer, spun down, resuspended in complete RPMI medium, and overlaid on 40%/80% (v/v) Percoll. Centrifugal condition was set at 600 × g for 20 min with no brake. The LPMCs were collected from the interface of gradient and incubated in complete RPMI medium.

### Flow cytometry and magnetic sorting

The following dyes and antibodies were used: SYTO BC dye (Invitrogen, 20 µM); PE-anti-mouse IgA mAb (4204, Thermo Fisher) or DyLight-550-IgA mAb (ab97000, Abcam); CD45.2 PerCP-Cy5.5 or FITC (0454, Thermo Fisher), CD11b APC (0112, Thermo Fisher), CD3 PE-Cy7 (0032, Thermo Fisher), CD19 APC (0193, Thermo Fisher), CD45R APC-eFluor 780 (or B220, 0452, Thermo Fisher), CD138 FITC (23554, Thermo Fisher), as well as anti-CD138 primary antibody (ab181789, Abcam) and FITC-conjugated Goat anti-Mouse secondary antibody (152312, Jackson). For magnetically sorting IgA-bound bacteria from fecal material, bacterial pellets were prepared with the method mentioned above, resuspended in 0.5 ml staining buffer, and stained with 10 µl PE-anti-IgA (4204, Thermo Fisher) for 30 min at 4°C. Stained bacteria were washed twice and resuspended in a 15 ml tube with 3 ml staining buffer. The sample tube was then placed on MACS special chill rack. The 2D code of anti-PE microbeads (048– 801, Miltenyi) was automatically recognized by the autoMACS Pro Separator (Miltenyi). The microbead reagent vial was assigned to special reagent rack slots. The cell labeling, separation and cleaning procedures were programed and automatically performed. IgA-bound bacteria were collected by eluting the positive fraction in MS column #1 and reloaded on MS column #2 for maximum purity. After determining the absolute bacterial count, samples were mixed with the cryoprotective agent and cooled at −80°C for future experiments.

### Western blot

Intestinal tissue lysate was prepared with ice-clod lysis buffer (Solarbio). The protein quantification of each tissue lysate was performed to determine loading quantity. The lysates were diluted with 2 × Laemmlin sample buffer and boiled to denature protein. Samples and molecular marker were loaded into a 12.5% SDS-PAGE gel for isolating desired protein. The protein was transferred from the gel to the activated PVDF membrane. The membranes were proceeded to blocking with blocking buffer, primary antibody incubation (ZO-1, 33–9100, Invitrogen; Occludin, 33–1500 Invitrogen; Claudin 3, ab214487, Abcam; β-actin, 4970, CST), washing and HRP-conjugated secondary antibody incubation (anti-rabbit IgG and anti-mouse IgG, EMD Millipore). Signal of the protein band was developed using ECL Substrate Kit (Abcam). All chemiluminescence images were acquired using Tanon 5200 Chemi-Imaging System (Tanon Science).

### IF and IHC

A 0.5 cm length of intestine tissue was placed into the embedding mold, filled with OCT in excess, and then a chuck was pressed over the mound with OCT overflown. The specimens were transferred into Cryostar N×50(Thermo Fisher) cryochamber at optimal freezing conditions after frozen completely in liquid nitrogen. The tissue was gently sliced into 5 µm thick sections and spread over the glass slide. The frozen sections were immediately dipped in acetone for rapid fixation. For cell slide, the cells grew on coverslips coated with polyethyleneimine and then fixed in purity methanol for 5 min. The sections were processed to antigen retrieval reagent (100 mM Tris, 4% urea, pH 6.0 or 9.5 for 10 min) and permeabilization (0.25% Triton X-100 in PBS for 10 min). Next, multicolor immunostaining was performed as following steps: blocking with PBST (0.1% Tween 20 and 10% FBS in PBS), diluted primary antibodies (MUC2, orb372331, Biorbyt; CD45, 13917, CST; CD138, ab181789, Abcam; IgA, ab97000, Abcam) incubation, secondary antibody incubation (Goat-anti-Rabbit IgG-Alex488, Goat-anti-Rabbit IgG-Alex555 and Goat Anti-Mouse IgG-Alex647, Abcam), nuclear staining with DAPI (8961, CST), and sealing coverslip with mounting medium. For IHC, the endogenous peroxidase activity of specimen was blocked with PBS containing 0.4% H_2_O_2_ for 10 min. Upon rinsing, slides were submerged in PBST for blocking. Diluted primary antibodies were applied to the section on the slide for 2 h in the dark. Slides were rinsed in PBS, incubated with biotinylated secondary antibody for 1 h and Sav-HRP conjugates for 30 min. DAB substrate was used for antibody color development. Then, slides were counterstained by immersing in hematoxylin for 2 min, dehydrated with alcohol, and sealed with mounting medium. Staining results were observed under an inverted confocal laser scanning microscope (Zeiss LSM 880, Germany) or SLIDEVIEW VS200 slide scanner (Olympus, USA).

### Ultrastructure imaging

A 0.5 cm portion of intestinal tissues of each sample was harvested and immediately immersed in fixative containing 3% glutaraldehyde, 2% paraformaldehyde and 4% sucrose for 6 h. Specimens were carefully cut into 3–4 mm^3^ without flushing intestinal content. Tissues were embedded within 3% agarose gel and trimmed into 5 mm^3^ gel-blocks. The gel-embedding effectively prevented bacteria adhering to the mucosa from being flushing away in the following processing. Gel- embedding tissue were proceeded to secondary fixation with 1.5% osmium tetroxide for 2 h. Fixed specimens were dehydrated by immersion into a gradually increasing concentration of ethanol. Following dehydration, tissue was placed into a 1.5 ml flat-bottom tube, infiltrated into liquid resin and cured into a hard block with heating. Tissues embedded in hardened resin were sliced into semi-thick sections (400 nm) and stained with 3% lead citrate and uranyl acetate. TEM were acquired with a Hitachi Model H-7650 (Hitachi).

### ELISAs

Serum, feces, and intestine samples were harvested from mice or piglets. All specimens were weighted, mashed, vortexed and homogenized in TBS (Tris, 150 Nm NaCl, pH 7.4) containing with protease inhibitors. The homogenates were spun at 2000 g for 20 min. the supernatants were collected and diluted prior to ELISA kit assays. Soluble immunoglobulin concentrations were measured according to the testing procedures of ELISA kits: mouse IgA (Bethyl Laboratories); pig IgA, IgG and IgM (Abcam).

### Statistics

Two-tailed Student’s *t*-test, one way-ANOVA or Mann–Whitney rank-sum test were performed with GraphPad software 8.0 or R statistical package. Data were presented as mean ± standard error of the mean (SEM). Significant differences were denoted by *p* < .05.

## Supplementary Material

Supplementary Materials.docx

## Data Availability

The raw sequencing data have been deposited in the National Center for Biotechnology Information (NCBI) Sequence Read Archive (SRA) repository with accession number PRJNA970096 and PRJNA974859. Source data are provided with this article. R markdown scripts enabling the analysis for data are uploaded at https://github.com/Tangwenjie34 and https://github.com/yusenWei. Specific codes are accessible from the authors. All database and detailed description of the methods are available in the main text or the supplementary materials.
