# Supplementary material for: IgA-mediated control of host-microbial interaction during weaning reaction influences gut inflammation
Source: Gut Microbes. 2024 Mar 4;16(1):2323220. doi: 10.1080/19490976.2024.2323220 (PMC10936605; doi:10.1080/19490976.2024.2323220)
Supplement: Supplementary Materials.docx [file KGMI_A_2323220_SM9298.docx]

Supplementary Materials for

**IgA-mediated control of host-microbial interaction during weaning reaction influences gut inflammation**

Wenjie Tang, Yusen Wei, Zhixiang Ni, Kangwei Hou, Xin M. Luo, Haifeng Wang Corresponding author: Haifeng Wang, [haifengwang@zju.edu.cn.](mailto:haifengwang@zju.edu.cn)

The PDF file includes:

**Supplementary Fig. S1 to S10**


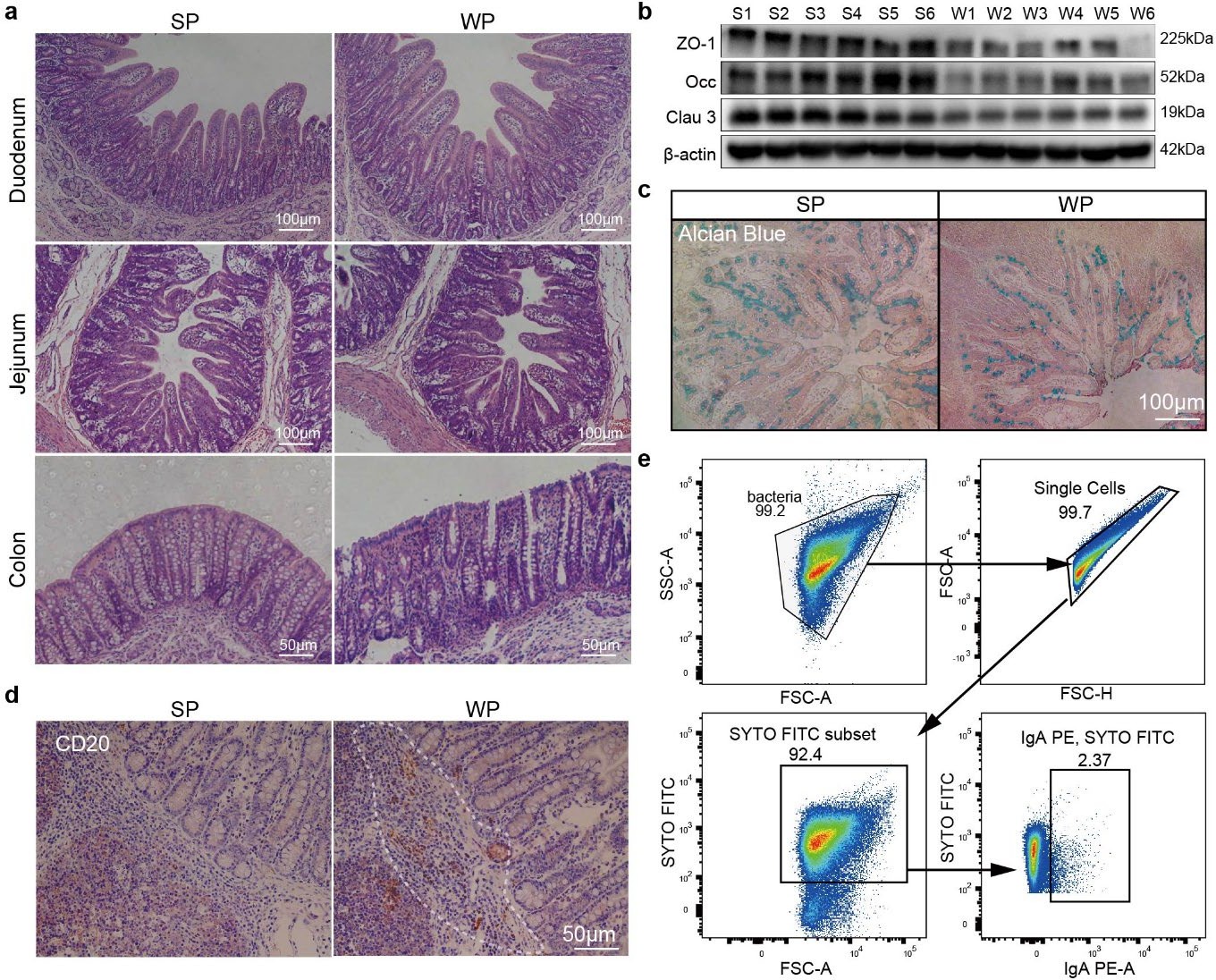


**Supplementary Fig. S1: Assessment of morphology and intestinal barrier function and gating strategy for detecting fraction of IgA+ bacteria in weaned piglets. a,** Representative micrograph of H&E-stained duodenum, jejunum, and colon sections from SP and WP (scale bar, 100 µm). **b,** Uncropped blots images of barrier function related protein form the overall sample. **c,** Representative images of Alcian blue/PAS-stained intestinal sections from the SP and WP groups (scale bars, 100 μm). **d,** Visualization of *CD20*+ B cell staining in intestinal tissue via representative IHC images of SP and WP. **e,** Representative flow cytometry gating strategy. Fecal samples were stained with SYTO-BC (bacteria) and anti-IgA-PE. Bacterially-sized events were initially gated based on FSC and SSC parameters. The selected events were subsequently subjected to further gating using FITC and PE channels to distinguish IgA+ and IgA- bacterial populations.


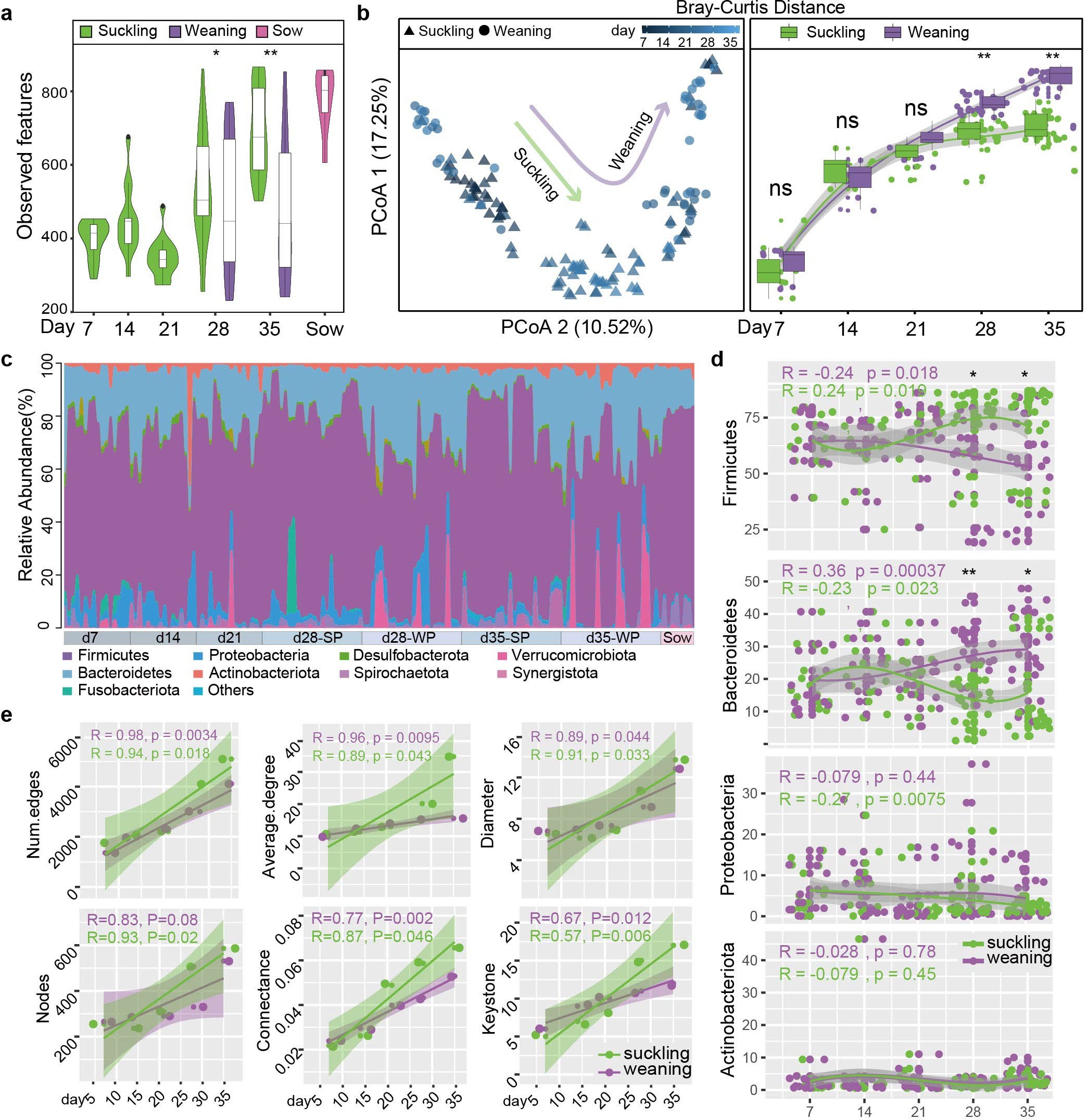


**Supplementary Fig. S2: The microbiome of piglets and its diversification corresponding to weaning process. a**, Total observed feature counts over time (One-way ANOVA). **b**, Principal coordinates analyses of weighed UniFrac distances of 16S community profiles of SP and WP groups (Mann-Whitney U test). **c**, Temporal dynamics shape the gut bacterial phyla composition of SP, WP and sow. **d**, Relative abundance of the Firmicutes, Bacteroidetes, Proteobacteria, and Actinobacteriota in SP (green) and WP (purple) groups. Adjusted R and *p* values from linear regressions were shown. **e**, Temporal changes of network topology, including number of edges, degree, diameter, number of keystone nodes, connectance, and the keystone. Adjusted R and *p* values from linear regressions are shown.


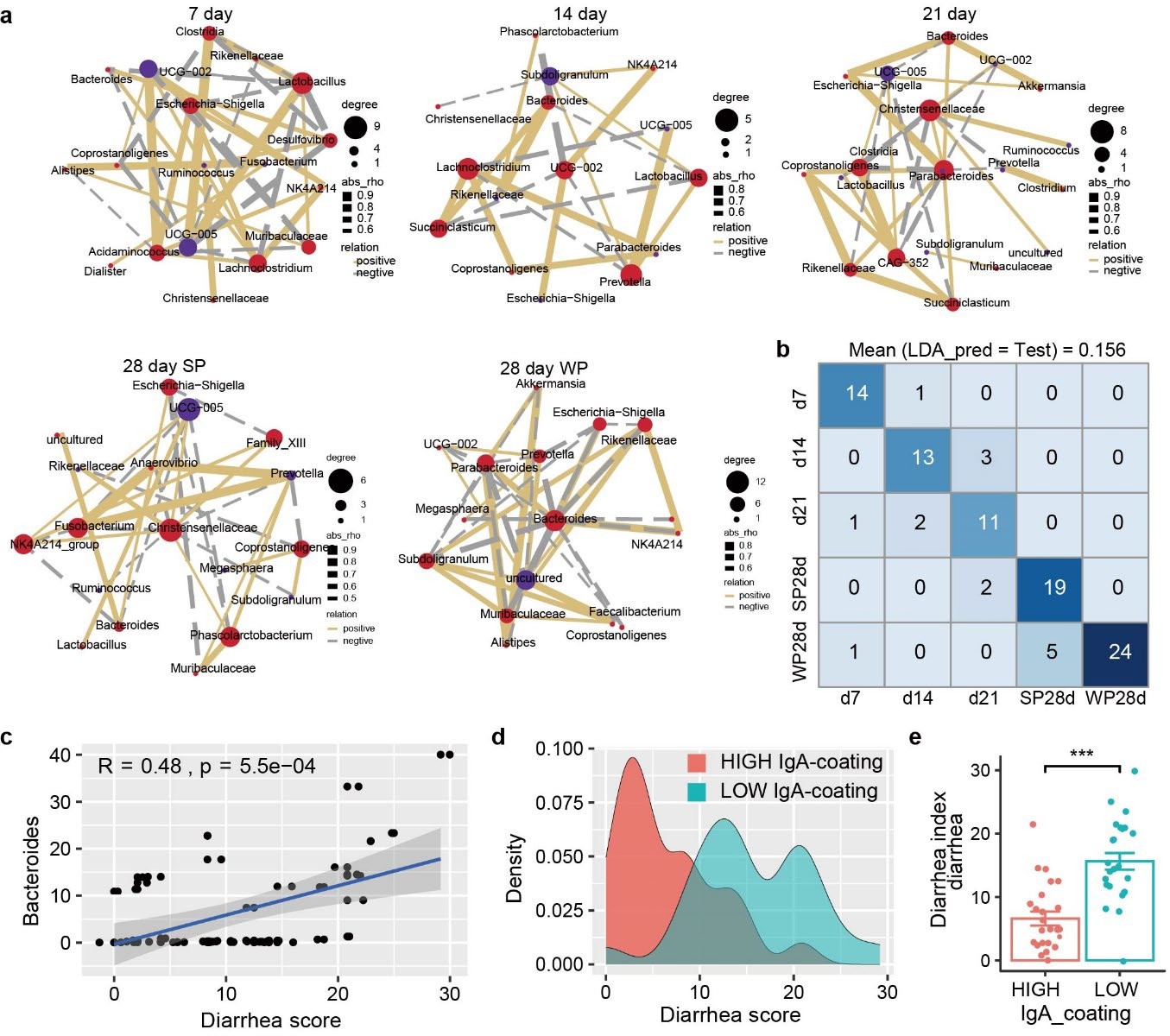


**Supplementary Fig. S3: Construction of Microbial co-occurrence patterns, Confusion matrix for LDA analysis, and grouping information. a**, Microbial co-occurrence patterns of days 7, 14, 21, 28 SP and 28 WP groups with fecal ecosystems were visualized using network diagrams Co-occurrence relationships with strong Spearman’s correlation coefficient |ρ value| > 0.5 and *p* < 0.05 were selected, the size of each node is proportional to the degree. **b**, Confusion matrix supported accuracy of the LDA predictive system in Fig. 2c. **c**, Relative abundance of the Bacteroides was positively correlated by linear regression with diarrhea score in all weaned piglets (n = 48). Linear regression and Pearson’s correlation coefficient were shown. **d**, Thresholding and assigning individuals into high IgA-coated bacteria group based on histogram of IgA-coated bacteria levels with threshold > 6.8. **e**, High IgA-coated bacteria group exhibits a significant decrease in diarrhea index compared to the low IgA-coated bacteria group in piglets. unpaired t test. ∗∗∗*p* < 0.001, mean ± SEM.


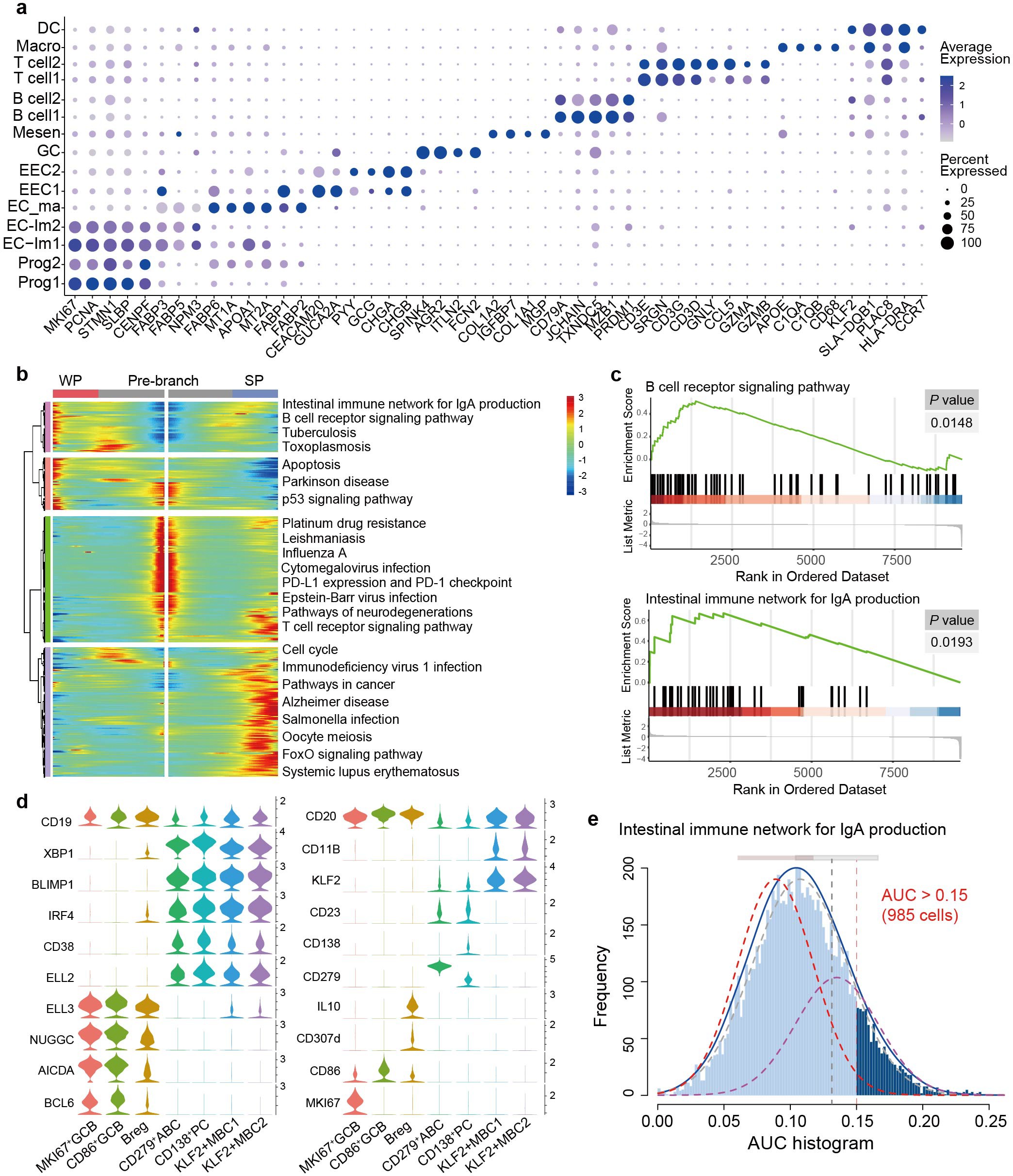


**Supplementary Fig. S4: Differential gene expression profiles. a,** Dot plot visualization of scaled expression levels of signature genes for each cluster subtype, annotated with colors corresponding to average gene expression. Dot sizes reflect the percentage of cells within each cluster expressing the respective gene. **b,** The differentially expressed genes (rows) along the branch (columns) of enterocytes into SP and WP clustering hierarchically into four profiles. The representative gene functions and pathways of each profile are shown. **c,** Enrichment score of B cell receptor signaling and IgA production pathways in enterocytes from SP and WP, according to the scRNA-seq data in Fig. 3F. **d,** Violin plots depicting the expression patterns of canonical marker genes across various B cell subtypes. **e,** Thresholding and assigning cells based on AUC Histogram of the gene set of the network for IgA production with threshold > 0.15.


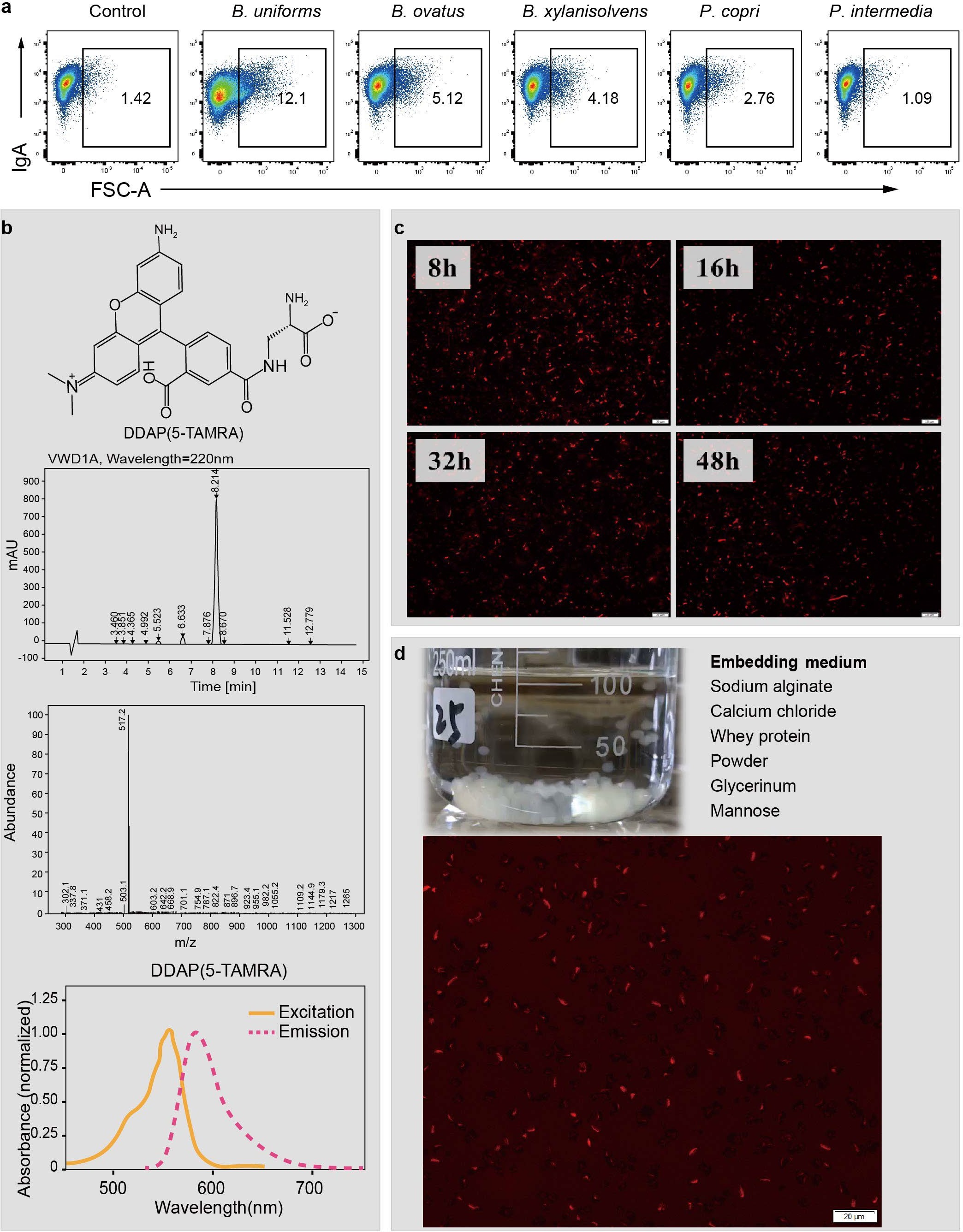


**Supplementary Fig. S5: Flow cytometry results relating to Fig. 5D and property of FDAA used in this study. a**, Representative flow cytometry plot of IgA-coated bacteria in the feces of mice mono- colonized with individual isolates. **b**, Structure and purity of the FDAA reported in this study, along with excitation and emission spectra of FDAAs in PBS at pH 7.4. **c**, Confocal images of *B. uniformis* stained with 5-TAMRA demonstrating less decay of fluorescent signal over 48 h. **d**, Retention of fluorescent signal by gel-embedded 5-TAMRA-labeled *B. uniformis* for over 24 hours *in vivo*.


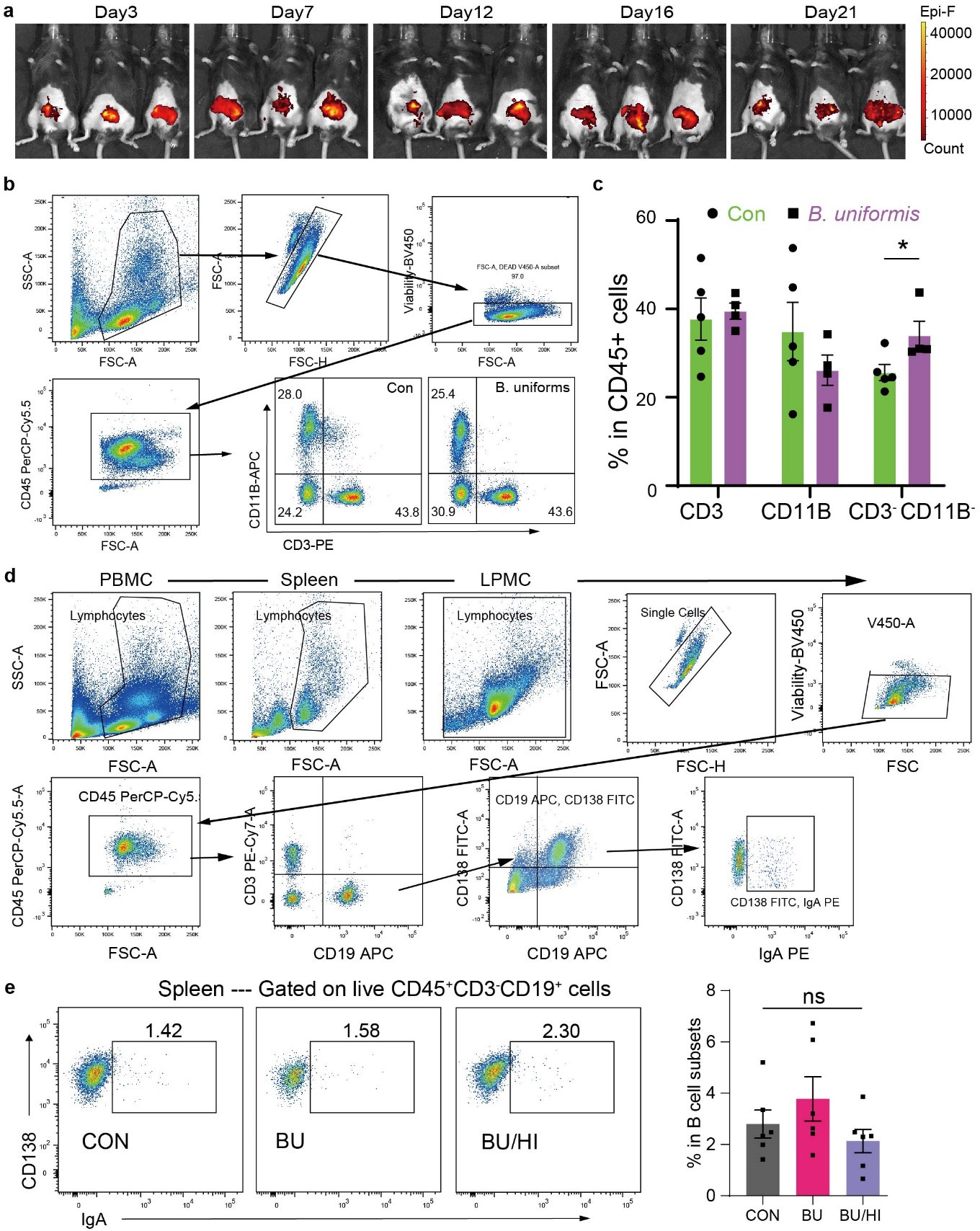


**Supplementary Fig. S6: Intravital imaging and flow cytometry gating strategy. a**, Assessment of the burden of FDAA-labeled *B. uniformis* in intact mice through whole-body imaging at days 3, 7, 12, 16, and

21. **b** and **c**, Gating strategy for flow cytometric analysis of intestinal immune cell subsets. Bar graph depicting the total cell counts of the indicated immune cell populations in Con- or *B. uniformis*-treated mice. Unpaired *t-*test, ∗*p* < 0.05, n = 6. **d**, Gating strategy for flow cytometric analysis of PBMC, spleen and LPMC *CD138*+ IgA+ PCs. **e**, spleens of all mice were collected for flow cytometry analysis of frequency of *CD138*+ IgA+ among live *CD45*+ *CD3*- *CD19*+ cells, with quantification of *CD138*+ IgA+ precent (n = 6). One way-ANOVA, ns, not significant, Mean ± SEM.


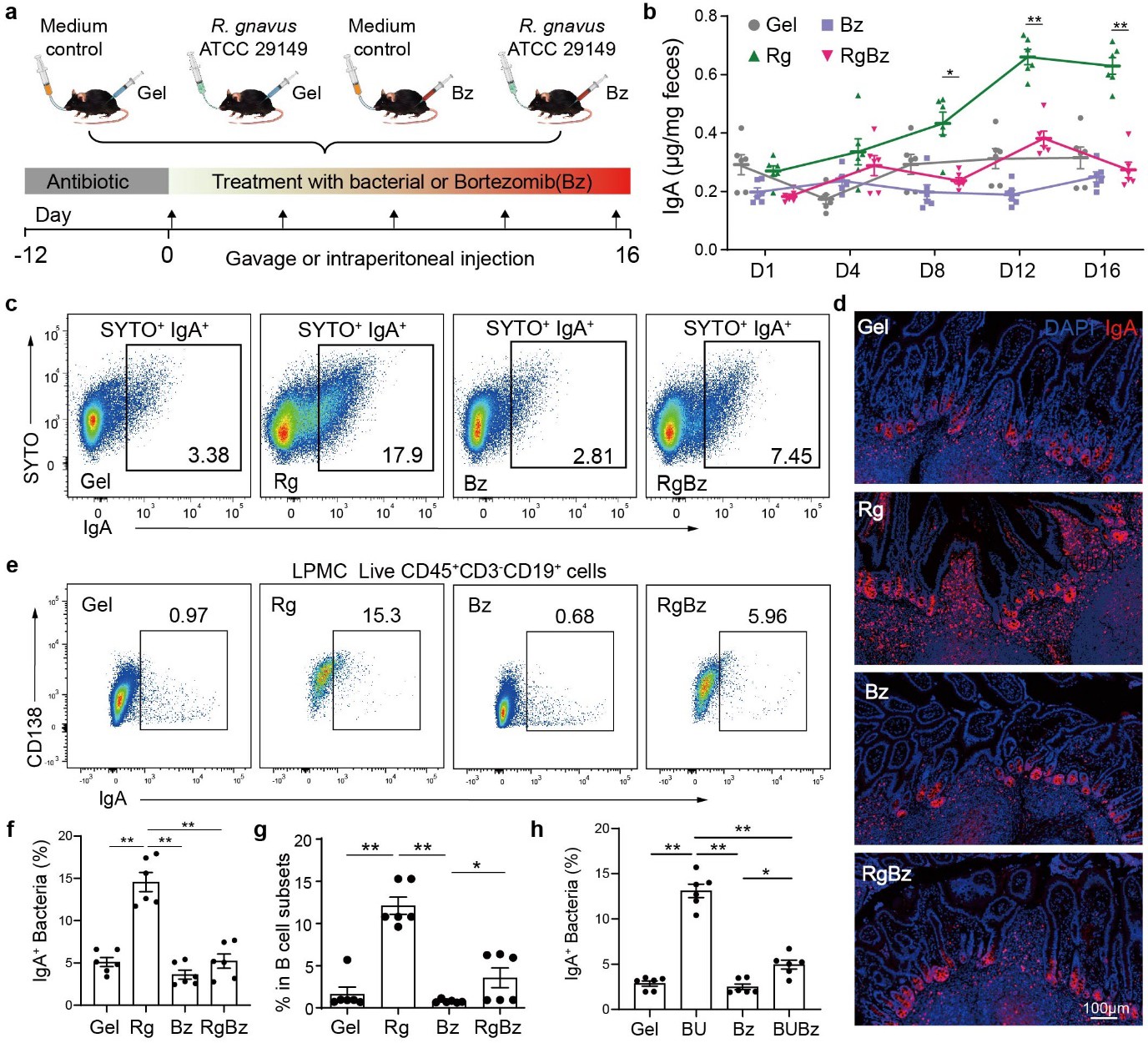


**Supplementary Fig. S7: Local effects of bortezomib (Bz)-mediated intestinal plasma cell depletion. a**, Schematic representation of intraperitoneal injection of either vehicle control hydrogel (Gel) or bortezomib-containing hydrogel (Bz) for the depletion of intestinal plasma cells. The positive control involved the use of *R. gnavus* ATCC 29249, a strain known to induce robust IgA responses in mice. **b**, ELISA for quantification of total fecal IgA levels in gel (control), Bz, *R. gnavus* mono-colonized, and *R. gnavus* + Bz-treated mice. One-way ANOVA, mean ± SEM, ∗*p* < 0.05, ∗∗*p* < 0.01, n = 6. **c**, Flow cytometry analysis of fecal bacterial IgA binding in gel (control), *R. gnavus*, Bz, or *R. gnavus* + Bz groups. **d**, Representative confocal images of IgA (red) immunolabelling in intestinal tissue, depicting the decrease of IgA+ cells in a mouse administered Bz (scale bar, 100 μm). **e**, LPMCs of all mice were collected for flow cytometry analysis of frequency of *CD138*+ IgA+ among live *CD45*+ *CD3*- *CD19*+ cells, with quantification of *CD138*+ IgA+ precents. **f**, Quantification of IgA-bound fraction related to Supplementary Fig. S8c. One way-ANOVA, mean ± SEM, ∗*p* < 0.05, ∗∗*p* < 0.01, n = 6. **g**, Quantification of *CD138*+ IgA+ fraction related to Supplementary Fig. S8e. One way-ANOVA, mean ± SEM, ∗*p* < 0.05, ∗∗*p* < 0.01, n = 6. **h**, Quantification of the IgA-bound fraction related to Fig. 6e. One way-ANOVA, mean ± SEM, ∗*p*

< 0.05, ∗∗*p* < 0.01, n = 6.


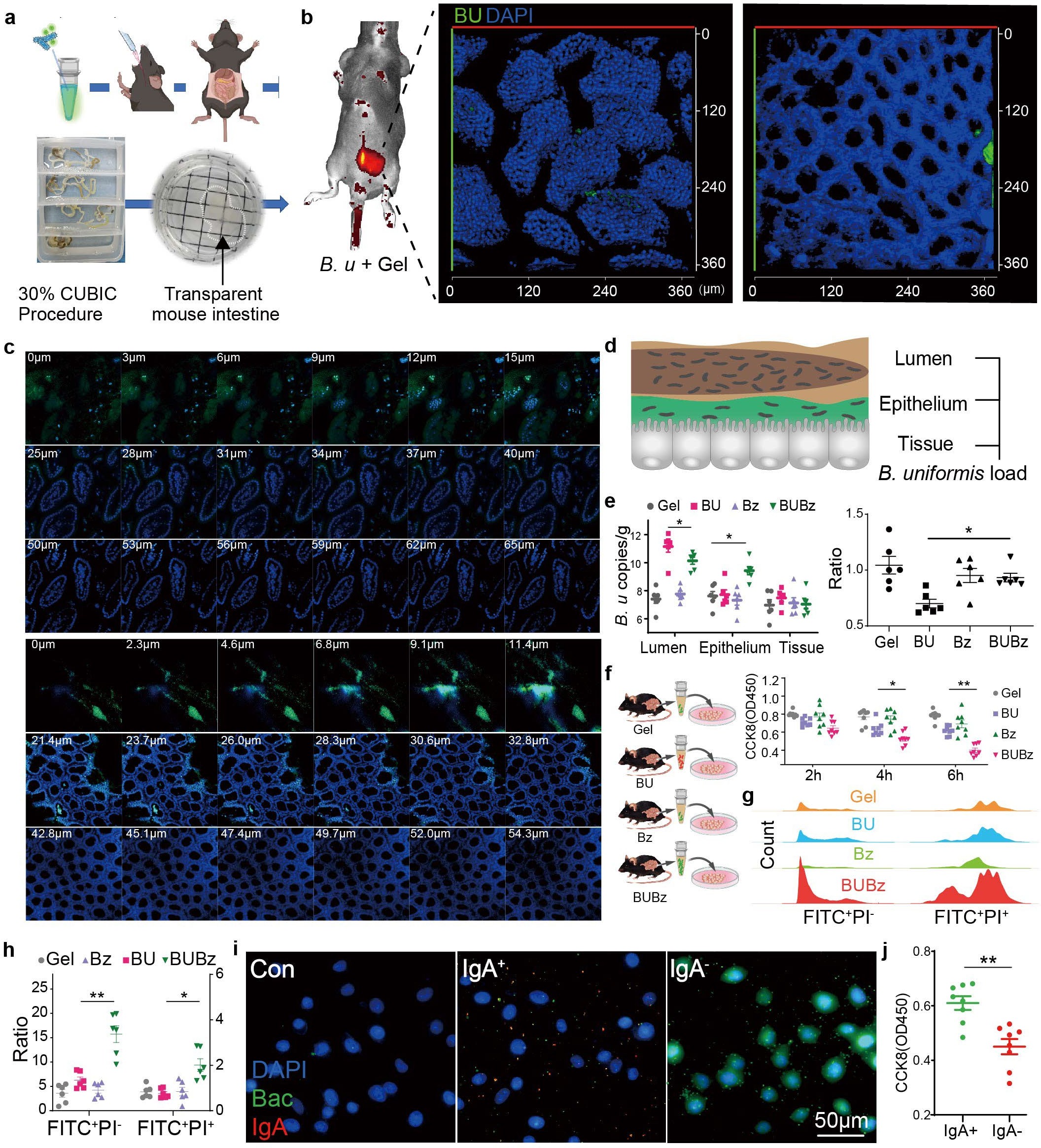


**Supplementary Fig. S8: *B. uniformis* traversed the lumen, interact with epithelial cells, and induced cell apoptosis when loss of IgA response**. **a** and **b,** Illustrative depiction of the labeling and imaging protocols employed for CUBIC-cleared intestine, alongside three-dimensional imaging of transplanted *B. uniformis* within the gut lumen. **c**, The acquired images of *B. uniformis* (green) and DAPI in a focal plane in different sections of ileum and colon. We performed the new high-speed imaging mode to record volumes of 800 × 600 × 200 μm3 with 41 image planes in 1.3 s (5-μm step size between adjacent planes), using an optical configuration providing a 3.00 ± 0.80-μm thick light sheet. **d**, Experimental framework for the analysis of *B. uniformis* load in the lumen, epithelium, and mucosal tissue by qPCR. **e**, *B. uniformis* load in lumen, epithelium, and mucosal tissue across groups (left). Ratio of *B. uniformis* load of epithelium/lumen from each group (right). **f**, Experimental schema of collecting fecal bacteria from gel (control), Bz, *B. uniformis*, and *B. uniformis* + Bz-treated mice and co-culturing with IPEC-J2 cells. The cell viability was determined by CCK8 assay. **g** and **h**, Cell apoptosis signal was detected by concurrent staining with Annexin V-FITC and PI. One way-ANOVA, mean ± SEM, ∗*p* < 0.05, ∗∗*p* < 0.01, n = 6. **i**, Confocal images of fixed IPEC-J2 cells for 6 h after inoculation of IgA+ and IgA- bacteria. Enlarged version of Fig. 6k. **j**, The cell viability of IPEC-J2 following inoculation with IgA+ and IgA- bacteria.

Unpaired *t*-test, mean ± SEM, ∗∗*p* < 0.001, n = 6.


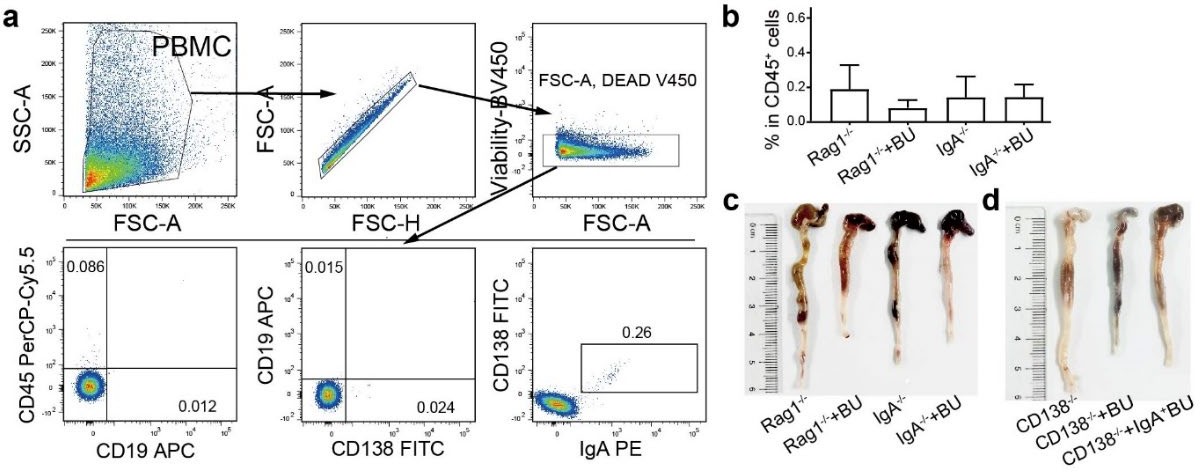


**Supplementary Fig. S9: Flow cytometry gating strategy and macroscopic image of the colon of gene knockout mice. a**, Gating strategy for flow cytometric analysis of *CD138*+ IgA+ PCs. The samples were collected from gene knockout mice. **b**, Quantification of *CD138*+ IgA+ PCs fraction in PBMC from *Rag*1-

/- or IgA-/- mice. One way-ANOVA, mean ± SEM, ns, not significant, n = 6. **c**, Macroscopic image of the colon harvested from *Rag*1-/- or IgA-/- mice. **d**, Macroscopic image of the colon harvested from *CD138*-/- mice.


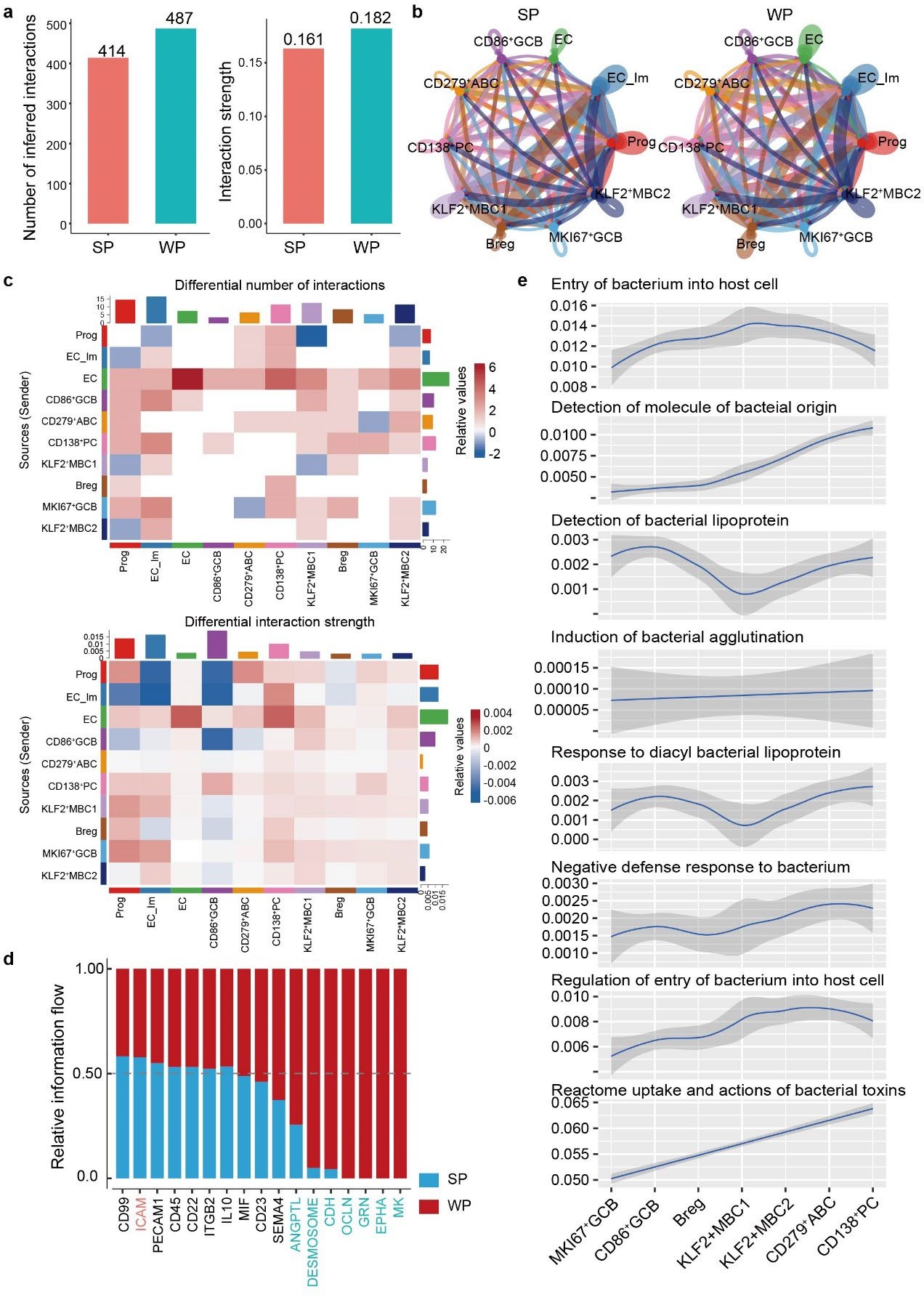


**Supplementary Fig. S10: Cell-cell and host-bacteria interactions. a**, Histogram showing total number of interactions and interaction strength in SP and WP groups. **b**, Circle plots showing the aggregated cell- cell communication network of interactions between any two clusters in SP and WP groups. Edge width is proportional to the number of interactions. **c**, The heatmap counting the number (up) and strength (down) of interactions between groups of cell clusters. **d**, The comparison of the signaling pathway based on the relative information flow between SP and WP groups. The top signaling pathways colored red are enriched in WP. **e**, Individual cell AUC score for host-microbiota interaction activities were subjected to statistical analysis, followed by liner regression analysis with the ranking of each B cell cluster.
